# Role of TET dioxygenases in the regulation of both normal and pathological hematopoiesis

**DOI:** 10.1186/s13046-022-02496-x

**Published:** 2022-10-07

**Authors:** Kanak Joshi, Lei Zhang, Peter Breslin S.J., Ameet R. Kini, Jiwang Zhang

**Affiliations:** 1 Department of Cancer Biology, Oncology Institute, Cardinal Bernardin Cancer Center, Loyola University Medical Center, Maywood, IL 60153 USA; 2 Cyrus Tang Hematology Center, National Clinical Research Center for Hematologic Diseases, Soochow University, Suzhou, 215123 China; 3 Departments of Molecular/Cellular Physiology, and Biology, Loyola University Medical Center and Loyola University Chicago, Chicago, IL 60660 USA; 4 Departments of Pathology and Radiation Oncology, Loyola University Medical Center, Maywood, IL 60153 USA

**Keywords:** TET2, Concurring mutations, HSPCs, Self-renewal, Differentiation, MDS, Leukemia

## Abstract

The family of ten-eleven translocation dioxygenases (TETs) consists of TET1, TET2, and TET3. Although all TETs are expressed in hematopoietic tissues, only *TET2* is commonly found to be mutated in age-related clonal hematopoiesis and hematopoietic malignancies. *TET2* mutation causes abnormal epigenetic landscape changes and results in multiple stages of lineage commitment/differentiation defects as well as genetic instability in hematopoietic stem/progenitor cells (HSPCs). *TET2* mutations are founder mutations (first hits) in approximately 40–50% of cases of *TET2*-mutant (*TET2*^*MT*^) hematopoietic malignancies and are later hits in the remaining cases. In both situations, *TET2*^*MT*^ collaborates with co-occurring mutations to promote malignant transformation. In *TET2*^*MT*^ tumor cells, TET1 and TET3 partially compensate for TET2 activity and contribute to the pathogenesis of *TET2*^*MT*^ hematopoietic malignancies. Here we summarize the most recent research on TETs in regulating of both normal and pathogenic hematopoiesis. We review the concomitant mutations and aberrant signals in *TET2*^*MT*^ malignancies. We also discuss the molecular mechanisms by which concomitant mutations and aberrant signals determine lineage commitment in HSPCs and the identity of hematopoietic malignancies. Finally, we discuss potential strategies to treat *TET2*^*MT*^ hematopoietic malignancies, including reverting the methylation state of TET2 target genes and targeting the concomitant mutations and aberrant signals.

## Key points

1. TETs regulate dioxygenase activity-dependent DNA demethylation and dioxygenase activity-independent histone modification.
2. TETs control the dynamic differentiation and lineage commitment of HSPCs by regulating the access of key transcription factors to the enhancers of target genes.
3. Somatic mutations of *TET2* are commonly detected in age-related clonal hematopoiesis and multiple types of hematopoietic malignancies.
4. Mutant *TET2* causes a pre-malignant condition by disrupting the epigenetic landscape and fostering genomic instability.
5. Mutant *TET2* collaborates with additional genomic mutations to induce hematopoietic malignancies.
6. *TET2-*mutant hematopoietic malignancies can be targeted pharmacologically by either restoration of dioxygenase activity or inhibition of dioxygenase activity. They can also be targeted clinically by combining demethylating agents with inhibitors of concurrent mutation-related signaling.

## Introduction

The multiple stagesof lineage commitment and differentiation processes of hematopoietic stem and progenitor cells (HSPCs) during both hematopoietic development and regeneration are tightly controlled by transcriptional machinery that is finely regulated by the stepwise reconfiguration of the DNA methylome and also by histone modifications [1, 2]. The ten-eleven translocation (TET) family of dioxygenases consists of TET1, TET2, and TET3. All three TET proteins catalyze the dynamic DNA demethylation process by converting 5-methylcytosine (5mC) to 5-hydroxymethylcytosine (5hmC), and further oxidizing 5hmC to 5-formylcytosine and 5-carboxylcytosine [3, 4]. TET proteins also regulate histone modifications including H3K4 methylation, H3K27 acetylation, and H2B monoubiquitylation by recruiting Set1/COMPASS and PRC1/2 complexes, all independent of their enzymatic activities [4–6]. TET proteins play such roles by collaborating with lineage-specific transcription factors (TFs), which determine the site-specific reconfiguration of the epigenetic landscape [7–9].

The methylation state of DNA sequences and methylation/acetylation/ubiquitination states of histone molecules in nucleosomes regulate the accessibility for TFs, specifically for methylation-sensitive TFs, to the regulatory elements of target genes, including their promoters and enhancers [10]. The cell-type-specific pioneer TFs can bind to methylated DNA and initiate lineage commitment and differentiation of HSPCs by recruiting TET proteins to enhancers/promoters of target genes to regulate the epigenetic landscape for the binding of methylation-sensitive TFs. In addition, the intermediate product of demethylation, 5hmC, can be recognized by specific TFs including MeCP2, the MBD3/NURD complex, UHRF1, UHRF2, SALL1/SALL4, PRMT1, RBM14 and WDR76 to induce target gene expression [11, 12]. It was reported that 5hmC is a critical mark of enhancer/promoter activation [13]. Moreover, TET proteins mark the sites of DNA damage and promote stability of the genome by regulating the ratio of 5hmC/5mC at gene body regions [14] and controlling the expression of DNA repair genes, including RAD50, BRCA1, RAD51, BRCA2, and TP53BP1 [15]. Thus, TET proteins function as tumor repressors in most types of hematopoietic malignant conditions.

In HSPCs, TETs, especially TET2, play critical roles in the regulation of the epigenetic landscape and control dynamic phases of lineage commitment at multiple differentiation stages [16–20]. Loss-of-function mutations of *TET2* (*TET2*^*MT*^) are frequently detected in small clones of hematopoietic cells in healthy persons, especially those > 50 years old. The frequency of such mutations is increased during aging, reaching ∼50% by 100 years of age, and has been named age-related clonal hematopoiesis (ARCH) or clonal hematopoiesis of indeterminate potential (CHIP) [21–25]. While cases of ARCH with *DNMT3A* mutation and *TP53/PPM1D* mutation display a growth advantage upon treatment with interferon-γ (IFN-γ) [26] or chemotherapeutic drug treatment [27], respectively, ARCH with *TET2*^*MT*^displays a growth advantage when treated with tumor necrosis factor-α (TNF-α) or interleukin 6 (IL-6) [6, 28–30]. Thus, ARCH might occur because of compensatory hematopoiesis against the increased inflammatory pressure of ageing and infection. Individuals with ARCH showed a 10–12 fold increased risk for the development of hematopoietic malignancies compared to age-matched ARCH-negative populations [31–34]. Consequently, *TET2*^*MT*^ is frequently detected in almost all types of hematopoietic malignancies. An accumulation of additional mutations promotes the malignant transformation of mutant HSPCs through collaboration with *TET2*^*MT*^. However, the mechanisms by which the additional mutations promote disease progression and determine disease identities of *TET2*^*MT*^ clones are only beginning to be clarified. In addition, in approximately 50% of *TET2*^*MT*^ hematopoietic malignancies, *TET2*^*MT*^are later hits [35, 36]. Studies suggest that the clinical features of hematopoietic malignancies with *TET2*^*MT*^ as a first or later hit are not the same [37]. Understanding the molecular processes that underlie such phenomena will help to enhance our ability to treat *TET2*^*MT*^ hematopoietic malignancies.

Although mutations of *TET1* and *TET3* are rare in hematopoietic malignancies, changes in *TET1* and *TET3* expression might be involved in the pathogenesis of *TET2*^*MT*^disease by partially compensating for the loss of TET2 [38, 39]. Thus, a better understanding of the molecular mechanism by which TET2 regulates normal hematopoiesis in cooperation with TET1/TET3, and the way *TET2*^*MT*^ is involved in the development and progression of hematopoietic malignancies together with other co-occurring mutations, can help in the development of novel pharmacological approaches to the treatment of *TET2*^*MT*^ diseases.

In this review, we summarize the recent research on the TET family of enzymes in regulating both normal and disease hematopoiesis, elaborate on the molecular mechanisms by which *TET2*^*MT*^ drives hematopoietic malignancies in combination with other genetic mutations, and discuss the potential for targeted therapy against *TET2*^*MT*^ malignancies.

### Role of TET dioxygenases in the regulation of normal hematopoiesis

The role of Tet proteins in the regulation of both embryonic hematopoietic generation and postnatal hematopoietic regeneration has been studied through the use of knockout animal models. Ablation of *Tet1*, *Tet2,* or *Tet3* individually leads to a modest decrease in 5hmC levels in the bone marrow (BM). The significant reduction of 5hmC levels in the BM of *Tet1*/*Tet2* compound knockout mice (*Tet1*^−/−^*Tet2*^*−/−*^) and *Tet2*/*Tet3* compound knockout mice (*Tet2*^−/−^*Tet3*^*−/−*^) suggests that *Tet1*, *Tet2,* and *Tet3* play certain redundant roles in the hematopoietic system and can compensate for one another. *Tet1*^−/−^*Tet2*^*−/−*^ and *Tet2*^−/−^*Tet3*^*−/−*^ HSPCs also show unrepaired DNA damage and impaired DNA repair, suggesting that Tet2 cooperates with Tet1 and Tet3 in maintaining genomic stability [20, 40–42].

#### Expression of Tet genes in normal hematopoietic tissues

All three Tet proteins are expressed in hematopoietic tissues, the expression of Tet1 being the lowest, as determined by RT-PCR [16, 43–45]. Tet1 is highly expressed in early HSPCs including long-term hematopoietic stem cells (HSC), multi-potent progenitors (MPP), lymphoid-primed multipotent progenitors (LMPP), common lymphoid (CLP) and myeloid progenitors (CMP), but is decreased during B lineage commitment/differentiation and is further reduced in erythroid progenitors (MEP), granulocyte and monocyte progenitors (GMPs), and megakaryocytes; it is undetectable in immature and mature myeloid cells [44]. One study suggested that the expression of Tet2 is higher than that of Tet3 [16]; however, another study presented the opposite result [45]. Tet2 is ubiquitously expressed in the hematopoietic compartment, including in all HSPC subsets and mature myeloid and lymphoid cells with reduced levels in MEPs with the lowest levels observed in Ter119^+^erythrocytes [43]. Tet3 is also ubiquitously expressed in the hematopoietic compartment, with the highest levels being observed in HSPCs; such levels are reduced during differentiation [45]. Knockout studies suggested that Tet2 accounts for nearly 60% of DNA dioxygenase activity in HSPCs, stressing the essential role that Tet2 plays in the regulation of normal hematopoiesis [46]. However, Tet1 and Tet3 might compensate for some dioxygenase activities under conditions of Tet2 loss in hematopoietic tissues and may contribute to the abnormal hematopoiesis observed in *TET2*^*MT*^ individuals and *Tet2*-knockout animals (*Tet2*^*−/−*^).

All three *Tet* genes also produce short isoforms as a result of the use of alternative promoters and splicing sites. The short isoforms of Tet2 (including Tet2a and Tet2c) lack catalytic domains and might function as dominant-negative inhibitors of the long isoforms. Both full-length Tet2 and its shorter truncated isoform Tet2a can be detected in hematopoietic cells. The expression of full-length Tet2 is higher than that of Tet2a [16]. The short forms of Tet1 (Tet1s) and Tet3 (including Tet3s and Tet3o) lack CXXC domains [47, 48]. The expression of shorter isoforms of Tet1 and Tet3 has not been examined in hematopoietic tissues. Due to their similar catalytic activity, the three Tets and their isoforms might have some redundant functions. However, owing to their different binding affinities to different genomic regions and different partner proteins, the three Tet proteins have distinct functions [49]. Even for individual Tet entities, the long and short isoforms have distinct or even opposite functions [50]. Thus it is very important to consider these isoforms when studying the functions of the Tet proteins.

#### The role of Tet proteins in early hematopoietic generation in the embryo

In the zebrafish embryo, both *Tet2* and *Tet3* are highly expressed in hemogenic endothelial cells (ECs) and are required for definitive HSC emergence, but not for the initiation of primitive hematopoiesis. Before HSC emergence, Tet2/3 regulates Notch signaling in the hemogenic endothelium, promoting the endothelium-to-HSC transition. Restoration of the Gata2b/Scl/Runx1 transcriptional network can rescue HSCs in *Tet2/3*double-mutant larvae [51]. In mice, all 3 Tets are expressed in early hematogenic tissues. *Tet2* and *Tet3* levels are induced during E7.5-E11.5 embryonic development, while *Tet1* expression is maintained at similar levels [52]. Combined loss of *Tet1* and *Tet2* does not impair embryonic hematopoiesis [53]. *Tet3*-knockout mice die perinatally without obvious hematopoietic defects [54]. Mice with combined loss of all 3 Tets are early embryonic lethal (E7.5-E8.5) due to defects in gastrulation [55]. To study the role of Tets in embryonic hematopoiesis, Ma et al. [52] generated mice with inducible loss of all 3 *Tets*. Induced deletion of all 3 *Tets* either globally or endothelial-specifically after gastrulation (E6.5–7.5) leads to reduced numbers of HSPCs and lethality at E11.5-E12.5 due to defects in the transition of ECs to HSPCs. Both primitive and definitive hematopoiesis are compromised in the mutant embryos which are associated with hypermethylation and down-regulation of *NFκB1, Gata1/2, Runx1* and *Gfi1b* genes in ECs. Re-expression of these genes can largely restore hematopoiesis in the knockout embryos as demonstrated in an explant culture system. However, distinct from the results of zebrafish studies, Notch signaling is not affected in the mutant mouse embryos, suggesting a Notch-independent mechanism of hematopoietic development. Consistent with what is observed with deletion of *Tets* in adult HSPCs (see below), *Tet*-deficient embryonic HSPCs exhibit a subtle lineage bias in colony formation assays but form aggressive myeloid malignancies in transplantation recipients. In human embryonic stem cells (ESCs), deletion of all *3 Tets* blocks formation of hematopoietic cells during the differentiation of ESCs to embryonic bodies, which is correlated with reduced expression of the master hematopoietic-specific transcription factors. These studies suggest a critical role of *Tets* in regulating of the emergence of both primitive and definitive hematopoiesis through regulating the expression of the master hematopoietic transcription factors. Nevertheless, the embryonic hematopoiesis in *Tet1* + *Tet3* compound-knockout mice and *Tet2* + *Tet3* compound-knockout mice has not been analyzed. Thus, whether all 3 *Tets* are required for the development of embryonic hematopoiesis remains to be determined.

#### The role of Tet2 in normal adult hematopoiesis

**Fig. 1:**
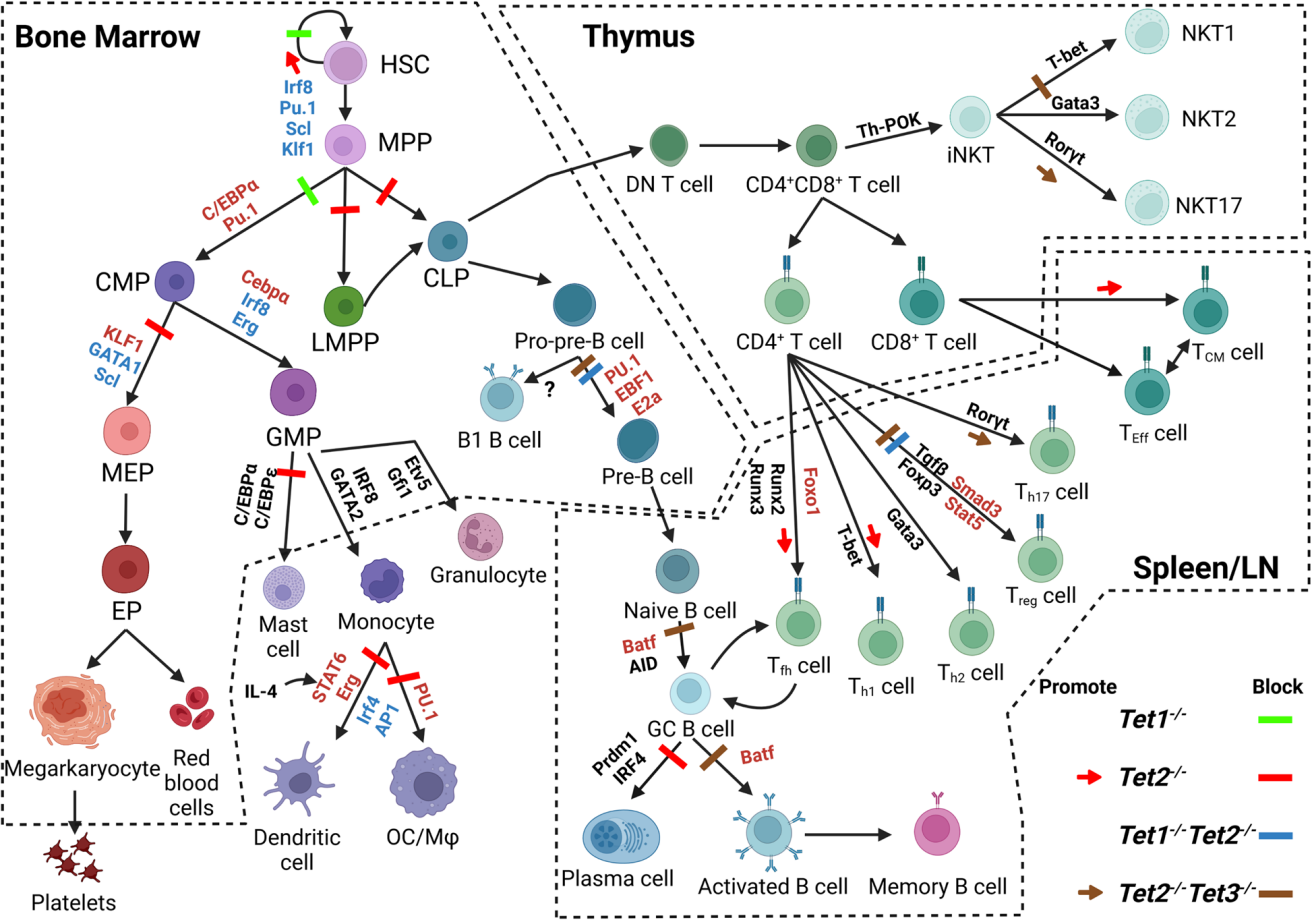
The roles of Tet proteins in normal and disease hematopoiesis as demonstrated by genetically-modified mouse models. Knockout mouse studies suggested that Tet2 regulates the dynamic differentiation and lineage commitment of HSPCs at multiple differentiation stages, including HSC-to-MPP differentiation, MPP-to-CLP, CMP-MEP, and CMP-GMP lineage commitments, pro-B-to-pre-B transition, GC B to plasma cells (PCs) vs. B1 B-cell lineage commitment, CD4 naïve T-to-Treg *vs*. Th17 and iNKT-to-NKT1 *vs*. NKT17 lineage decision, as well as CD8^+^ memory T cell generation. This explains the pleiotropic hematopoietic disease profile of *TET2*^*MT*^ malignancies. Tet1 antagonizes Tet2 activity in the regulation of HSC self-renewal and myeloid *vs*. B-cell lineage commitment. However, Tet1 collaborates with Tet2 in regulating immature B-cell-to-mature B-cell differentiation and naïve CD4^+^ T-to-Treg cell differentiation. Consequently, knockout of both *Tet1* and *Tet2* in HSPCs leads to B-ALL-like disease owing to the aberrant expansion of immature B-cells, while knockout of both *Tet1* and *Tet2* in CD4^+^ T or Treg cells, resulting in autoimmune/inflammatory disease due to impaired Treg cell production. However, Tet3 compensates for Tet2 activity in almost all types of cells studied. As a result, mice with *Tet2* and *Tet3* compound-deletion in 1) HSPCs develop AML within 1–3 months; 2) pro-B cells develop B-ALL within months; 3) immature B-cells develop lupus-like autoimmune diseases; 4) CD4^+^ T-cells develop PTCL with NKT17 phenotype, and 5) FoxP3^+^ Treg cells develop autoimmune lymphadenopathy. The TFs in red font are lineage-specific pioneer TFs that are required for recruiting Tet proteins to DNA for DNA demethylation, while the TFs in blue font are dependent on Tet2-mediated demethylation to access their target gene enhancers. The TFs in black font are dependent on Tet2 for their expression. (Created with BioRender.com)

Knockout mouse studies have demonstrated that Tet2 plays a key role in adult hematopoiesis (Fig. 1 and Table 1).

**Table 1:** Phenotypes of Tet1, Tet2, and Tet3 knockout mice and compound-knockout mice

Mouse lines	HSCs and MPPs	Latency	Hematopoietic diseases
*Tet1*^*−/−*^ mice [44]	Higher frequencies of immature B cells Increased self-renewal capacity and frequency of B-cell progenitors	18–24 months	B-cell lymphoma
*Tet2*^*−/−*^ mice [16, 30, 35, 43]	Expansion of LSK HSPCs and GMPs. Increased CHRC in competitive transplantation Dramatically increased proportions of Gr1^+^/Mac1^+^ cells in their BM, spleens, and PB after 1 year Genome-wide increase in DNA methylation of active enhancers and downregulation of genes including *Gata2*	12–20 months	Develop microbial-induced MDS/MPNs and CMML in > 90% of the animals at 12–15 months Approximately 4–10% of animals develop B-cell malignancies or T-cell malignancies
*Tet3*^*−/−*^* mice*		Die at birth	
*Vav1*^*Cre*^*Tet3*^fx/fx^ mice	A minor increase in the frequency of LSK HSPCs and a decrease in the frequency and absolute numbers of HSCs in BM		Healthy
*Vav1*^*Cre*^*Tet2*^fx/fx^ mice *Mx1*^*Cre*^*Tet2*^fx/fx^ mice	Dramatically increased proportions of Gr1^+^/Mac1^+^ cells in BM, spleen, and PB	12–14 months	CMML like
*Tet2*^*KD*^* mice * [56] *(H1367Y/D1369A)*	Slightly increased LSK HSPCs, significant expansion of, CMPs and GMPs Distinct gene expression pattern compared to *Tet2*^*−/−*^	Starting at 4 months 12–20 months	Predominantly MDS/CMML
*LysM*^*Cre*^*Tet2*^fx/fx^ mice [48]			Healthy
*Tet2*^*gt*^ mice (gene trap at the 2^nd^intron) causes an 80% decrease in Tet2 mRNA levels and a 50% decrease in 5hmC levels [57].	Cooperate with TCR signaling to decrease FoxO1 expression and activity	∼17 months	Lymphoproliferation of Tfh-like cells
*Vav*^*Cre*^*Tet2*^fl/fl^ OT-II T-cell receptor transgene [58]		10 months	Developed AITL-like lymphomas
*CD4*^*Cre*^*Tet2*^fx/fx^[59]	Minimal effect on thymic T-cell development		Increased CD8^+^ memory T-cells after viral infection, improved protection upon subsequent re-infection
*Cd19*^*Cre*^*Tet2 *^*fx/fx*^* mice * [60–62]	Germinal center (GC) hyperplasia impairs plasma cell differentiation and promotes B-cell lymphomagenesis Increase in AID-mediated mutations GC B-cell hyperplasia and impaired plasma cell differentiation Decreased expression of Prdm1	16 months	CLL-like Precipitated malignancy induced by T-cell leukemia/lymphoma 1A (TCL1A) Δexon 3
*CD19*^*Cre*^*Tet2*^fx/fx^*Tet3*^fx/fx^ [63]	Hyperactivation of B- and T-cells, autoantibody production Downregulation of *CD86*		Lupus-like disease
*Mb1*^*Cre*^*Tet2*^fx/fx^*Tet3*^fx/fx^ [40, 64]	Block at the transition from the pro–B-cell to the pre–B-cell stage Down-regulation of IRF4 Increased CpG methylation at the Igκ 3' and distal enhancers, influencing chromatin accessibility of B-cell-specific TFs such as E2A or PU.1	5–6 months	Developed B-cell lymphomas with splenomegaly and lymphadenopathy. Resemble human B-ALL
*Mx1*^*Cre*^*Tet2*^fx/fx^*Tet3*^fx/fx^ [20] *Mx1*^*Cre*^*Tet2*^fx/+^*Tet3*^fx/fx^ [65] *Mx1*^*Cre*^*Tet2*^fx/fx^*Tet3*^fx/+^	Uncontrolled expansion of CD11b^+^Gr1^+^ immature monocyte/granulocytes Tet2 and Tet3 are dose-dependent	1–3 months 5–10 months	AML
*Tet1*^−/−^*Tet2*^−/−^ mice [41]	Increases CLP/BLP compartment and affects B-cell development HSCs exhibit an increased short-term, but not long-term, hematopoietic repopulating capacity Express genes of human B-cell malignancies such as *Lmo2* genes of Bcl6, *Myc*, *Pten*, and *Blk*	15–20 months	B -ALL
*Tet2*^−/−^*CD4*^*Cre*^*Tet3*^fx/fx^ [17]	iNKT cells skew toward the NKT17 lineage, stimulated by TCR signaling	2 months	Aggressive PTCL-like syndrome originating from iNKT cells. CD1d-restricted iNKT cell lymphoma
*Foxp3*^*Cre*^*Tet2 *^*fx/fx*^*Tet3 *^*fx/fx* ^ [66]	Hypermethylation at *FoxP3* promoter and intronic enhancer *CNS2*, impaired Treg cell differentiation and function	1 month?	Develop autoimmune disease Develop inflammatory disease
*Tet1*^*−/−*^*Cd4*^*Cre*^*Tet2 *^*fx/fx* ^ [67] *Tet1*^*−/−*^*Foxp3*^*Cre*^*Tet2 *^*fx/fx*^	H2S regulates *Tet1* and *Tet2* expression via sulfhydration of NFYB CD4^+^ cells show strong skewing towards Tfh/Th17 phenotypes Hypermethylation at *FoxP3* promoter and intronic enhancer *CNS2*, impaired Treg cell differentiation and function		Develop autoimmune disease

Germline *Tet2* knockout mice (Tet2^*−/−*^) are viable and fertile [16, 43, 68]. *Tet2* deficiency in HSCs results in expansion of HSCs and GMPs and a reduction of MEPs and CLPs, as demonstrated in a study of germline *Tet2* knockout, *Mx1*^*Cre*^*Tet2*^*fx/fx*^ and *Vav*^*Cre*^*Tet2*^*fx/fx*^ mice. *Tet2*^*−/−*^ mice showed myeloid-biased hematopoiesis as demonstrated by the expansion of granulocytes/monocytes and reduced numbers of T/B lymphocytes [19, 69, 70]. Competitive hematopoietic reconstitutive capacity (CHRC) of *Tet2*^*−/−*^ BM hematopoietic cells is significantly enhanced as demonstrated by competitive BM cell transplantation and serial transplantation assays, suggesting enhanced self-renewal of HSCs [19, 69, 70]. However, a purified HSC transplantation study suggested that the enhanced CHRC of *Tet2*^*−/−*^ BM hematopoietic cells is not due to the enhanced self-renewal of HSCs but rather to the increased proliferation of mutant CMPs and GMPs [71]. *Tet2*^*−/−*^ mice developed pleiotropic hematopoietic abnormalities: > 90% of mice developed chronic myelomonocytic leukemia (CMML)-like myeloid proliferative neoplasms (as demonstrated by expansion of myelo-monocytic progenitors and monocytes) while the remaining mice developed chronic lymphocytic leukemia (CLL)-like diseases after long-term latency [16, 30, 35, 43]. *Tet2*^*−/−*^ mice develop peripheral T-cell lymphoma (PTCL)-like diseases with active antigen stimulation at 10 months of age [58]. Thus, Tet2 is a critical tumor suppressor in hematopoietic tissue. Tet2 plays such a role primarily by regulating the dynamic DNA demethylation and chromatin modifications at enhancers and promotors of key genes that determine lineage commitment and differentiation in HSPCs [18]. Loss of *Tet2* leads to DNA hypermethylation of active enhancers, which represses the access of the key TFs, including Gata2, for lineage commitment and differentiation. Tet2 is also involved in maintaining genomic stability by regulating the 5hmC/5mC ratio at gene body regions, thereby restricting gene mutations. Consequently, *Tet2* loss leads to hypermutagenicity in HSPCs [42]. In addition, Tet2 also has a poorly defined catalytic-independent activity that regulates HSC self-renewal but not myeloid lineage commitment [16, 35]. A comparative study of *Tet2*^*−/−*^ and *Tet2*^*KD*^(catalytic activity dead) mice suggested that the myeloid biased feature is due to the loss of catalytic activity while the expansion of HSCs is primarily mediated by a catalytic-independent mechanism [56].

Studies of linage specific-knockout mice suggest that Tet2 regulates dynamic lineage commitment and differentiation at multiple differentiation stages and represses leukemia/lymphoma development [18]. Distinct from mice with *Tet2*^*−/−*^ in HSPCs, *Tet2* deletion in differentiated myeloid cells (*LysM*^*Cre*^*Tet2*^fx/fx^ mice) is not sufficient to cause myeloid malignancy, suggesting a HSPC-specific phenotype. Detailed analyses demonstrated that the CMML-like disease in HSPC *Tet2*^*−/−*^ mice is stimulated by blood dissemination of intestinal microbes due to the dysfunction of the small-intestinal barrier [30]. Microbial contamination in the blood stimulates inflammation and increases IL-6 and TNFα production, which in turn stimulate the aberrant expansion of myeloid cells [28, 29]. The IL-6/Shp2/Stat3 axis promotes the development of CMML-like disease by inducing Morrbid expression. These studies suggested that *Tet2*^*−/−*^ HSPCs gain a growth advantage and a myeloid bias in differentiation under infection and inflammatory stress. Interestingly, *Tet2* deletion in myeloid cells fails to induce such bacterial dissemination. Therefore, which types of *Tet2*^*−/−*^ HSPCs are responsible for the dysfunction of the small-intestinal barrier needs to be determined in the future.

Detailed mechanistic studies demonstrated that Tet2 is involved in the regulation of myelopoiesis at multiple differentiation stages. Tet2 does so by collaborating with master epigenetic pioneer TFs such as Pu.1 and Runx1, to reshape the genomic landscape of 5mC and 5hmC, which regulates the genomic motif accessibility of the key lineage specific TFs for the expression of genes involved in lineage commitment and differentiation as well as leukocyte function and immune response [72–74]. In HSCs, Tet2 functions differently from Dnmt3 in regulating myelo-monocytic *versus* erythroid progenitor differentiation by repressing genomic accessibility of the key myelo-monocytic TFs (*e.g.*, Irf8 and Pu.1) and promoting genomic accessibility of the key erythroid TFs (*e.g.*, Gata1, Scl and Klf1). *Hoxa9* and *Gata2* have been identified as Tet2 target genes in HSCs which are involved in the regulation of lineage fate [56]. *Tet2*^*−/−*^ alters the genomic methylation landscape in HSCs and skews HSC transcriptional priming toward myelo-monocytic *versus* erythroid progenitor differentiation [75]. During the transition of MPPs to CMPs, Cebpα, in concert with Pu.1, recruits Tet2 to the regulatory regions of myeloid genes such as (Klf4, Chd7, Jun and Smad3) to establish the myeloid cell fate [74, 76]. During lineage commitment of CMPs to GMPs and MEPs, Tet2 regulates genomic accessibility of the master erythroid TFs (*eg.*, Gata1, Klf1 and Scl) and the master myeloid specific TFs (*eg.*, Cebpα, Irf8, Erg, and Runx1) for the monocytic/granulocytic and erythroid progenitor commitments [72, 73]. The Pu.1-Tet2 complex regulates the differentiation of GMPs to monocytes/macrophages and granulocytes [77–79]. Tet2 also regulates the GMP-to-mast cell differentiation by modulating the expression of Cebpα and Cebpε [80]. During monocyte-to-dendritic cell differentiation, IL4-Jak3-Stat6 induces the expression of Egr2 which then targets Tet2 to the transient binding sites of target genes (*eg.*, Batf3 and Irf4) to prime the differentiation process. Egr2 further coordinates with other TFs (*eg.*, Ifr4 and AP1), together with Tet2, to the stable binding sites to induce dendritic cell biology [78, 81–83]. During monocyte-to-osteoblast differentiation, Pu.1 recruits Tet2 to the promoters of key osteoclast-genes (*eg.*, Acp5, Ctsk, and Tm7sf4) [79]. (Figure 1) In addition, through interacting with IκBζ or Egr1, Tet2 represses the production of inflammatory cytokines IL-6 and MIF in monocytes by recruiting histone deacetylases to their promotors [6]. Tet2 also represses the production of IL-1β in monocytes through restraining the activity of IL-1β/NLRP3 inflammasomes [84]. Furthermore, Tet2 also restricts LPS-stimulated production of inflammatory cytokines such as IL-1β, IL-6 and Arginase 1 in macrophages [85]. These inflammatory cytokines not only promote the development of *Tet*^*mut*^myeloid malignancies via stimulating the proliferation and survival of mutant clones, but also contribute to the pathogenesis of several age-related pathologic conditions, including atherosclerosis, cardiovascular disease, and vascular complications [86].

Tet2 regulates pro-B-to-pre-B progenitor differentiation and B1 and B2 lineage commitment during the development of early lymphocytic progenitors. B-cell-specific *Tet2* knockout mice (*Cd19*^*Cre*^*Tet2*^fx/fx^) display an abnormal accumulation of CD19^+^ B220^low^IgM^+^IgD^−/low^CD43^+^CD21^−^CD23^−^Mac1^low^CD5^+^B1 like-cell populations and develop CLL-like malignancies after long latencies (> 16 months) [60]. In later stages of B-cell differentiation, Tet2 functions as a tumor suppressor for mature B-cell malignancies by regulating germinal center (GC) B-cell exit of the GC reaction and plasma cell differentiation [61]. *Tet2* deficiency in GC cells leads to GC hyperplasia and impaired plasma cell differentiation and predisposes to B-cell malignancies [61]. The B-cell malignancies in *Tet2*^−/−^mice depend on activation-induced deaminase (AID)-induced mutation for their development and BCR (B cell receptor) signaling for their survival [60]. The gene expression profile and DNA methylation signatures of *Tet2*^*−/−*^ GC B-cells is reflected in patients with *TET2*^*MT*^ diffuse large B cell lymphomas (DLBCLs). The conceptual similarity of *Tet2*^*−/−*^ GC B-cells to GC B-cells containing *KMT2D*, *CREBBP,* or *EP300* mutations suggests that Tet2 might collaborate with these histone modifiers in regulating target genes [61]. The plasma cell differentiation defects of *Tet2*^*−/−*^ GC B-cells are caused by the failure of upregulation of the plasma cell master regulators Prdm1 (Blimp1) and Irf4 (Fig. 1) [61]. PRDM1 loss occurs almost exclusively in patients with ABC-DLBCLs, many of which manifest a plasmablastic transcriptional profile.

In T-cells, TCR (T cell receptor) signaling rapidly and dynamically regulates Tet2 expression and activity. Tet2 regulates CD4^+^ T helper cell differentiation and CD8^+^ memory T-cell generation. T-cell-specific *Tet2* knockout mice (*CD4*^*Cre*^*Tet2*^fx/fx^) show minimal changes in T-cell development in the steady-state. However, after viral infection, more CD8^+^ memory T-cells are detected in *Tet2*^*−/−*^ mice, which are associated with improved protection upon subsequent re-infection [59]. The key TFs that mediate Tet2 function in the generation of central memory T-cells need to be identified. In the developing germ center during antigen-stimulated CD4^+^ naïve T cell differentiation, Tet2 is recruited to the regulatory locus of target genes by Foxo1 and Runx1 to restrict the lineage commitment of T follicular helper cells (Tfh) by facilitating the expression of negative regulators of these cells (*eg.*, Runx2 and Runx3) [9]. Thus *Tet2*^*−/−*^ CD4^+^ naïve T cells preferentially differentiate into Tfh cells.

Global 5-hmC profiling demonstrated that 5-hmC is significantly induced during human CD34^+^HSPC commitment to the erythroid lineage followed by a dramatic decrease throughout subsequent erythroid differentiation [87]. Such dynamic changes in 5-hmC profiling is associated with TET2 levels and activity that are induced by EPO-stimulated JAK2-TET2 phosphorylation [87, 88]. The locus-specific distribution of 5-hmC in erythroid progenitors is correlated to the specific binding of erythroid-specific TFs GATA1, GATA2, and KLF1 at promotors of erythroid genes such as the HB cluster genes [87]. The aberrant erythropoiesis was described in *Tet2*^*−/−*^ mice which recapitulated the ineffective and dysplastic erythropoiesis observed in MDS patients [43]. The frequencies of erythroid progenitors in BM are reduced in *Tet2*^*−/−*^ mice and are associated with a reduction of red blood cells in peripheral blood [75]. In zebrafish models, Tet2 plays an essential role in erythropoiesis by regulating the expression of the lineage-specific genes Scl, Gata-1 and Cmyb. Tet2 deletion leads to erythrocytic dysplasia and anemia which is associated with promoter hypermethylation of Scl, Gata-1 and Cmyb genes [89]. The role of TET2 in the differentiation of human erythroid progenitors has been studied in human CD34^+^ HSPCs in an in vitro setting. *TET2 *knockdown led to hyper-proliferation of CFU-E progenitors via upregulation of c-Kit, followed by expansion of a dysfunctional population of CFU-E cells via upregulation of AXL [90, 91].

#### Tet1 and Tet2 play lineage-specific compensatory or antagonist roles in adult hematopoiesis

Germline *Tet1* knockout mice (*Tet1*^*−/−*^) are viable and fertile [68]. The CHRC of BM cells from *Tet1*^*−/−*^ mice is reduced compared to wild-type (*WT*) controls, suggesting impaired self-renewal of *Tet1*^*−/−*^ HSCs. *Tet1*^*−/−*^ mice develop B-cell lymphomas with longer latencies (~ 18–24 months) [44] (Table 1).

Many *Tet1*^*−/−*^*Tet2*^*−/− *^mice die perinatally; the survivors are weaker and smaller and have reduced fertility [53]. Cells from *Tet1*^*−/−*^*Tet2*^*−/−*^ mice are hypermethylated with compromised imprinting. Detailed analysis demonstrated that Tet1 antagonizes Tet2 in the regulation of HSC self-renewal and malignant myeloid development but compensates for Tet2 in preventing B and T lymphocytic malignancies. The enhanced CHRC and myeloid-biased differentiation of *Tet2*^*−/−*^ HPCs can be attenuated by *Tet1* deletion [41]. *Tet1* loss impairs the enhanced self-renewal of HSCs and represses the expansion of GMPs in *Tet2*^*−/−*^ mice. *Tet1* deletion dramatically decreases the incidence and markedly delays the onset of *Tet2* deletion-related myeloid malignancies. *Tet1*^*−/−*^*Tet2*^*−/−*^ mice develop lethal B-cell malignancies at a later age. In T-cells, Tet2 stabilizes FoxP3 expression in Treg cells and regulates Treg activity in cooperation with Tet1. Deletion of *Tet1* and *Tet2* in T-cells (*CD4*^*Cre*^*Tet1*^*−/−*^*Tet2*^*fx/fx*^ and *Foxp3*^*Cre*^*Tet1*^*−/−*^*Tet2*^*fx/fx*^ mice) leads to hypermethylation of the CNS2 enhancer of the *FoxP3* gene and impaired Treg cell differentiation and function. T-cell-specific *Tet1* and *Tet2* knockout mice develop autoimmune diseases [67] (Fig. 1 and Table 1).

#### Tet3 compensates for Tet2 function in regulating the lineage commitment and differentiation of HSPCs at multiple differentiation stages

Germline *Tet3*^*−/−*^ mice can develop to term but die at birth [54]. HSC-specific *Tet3* mice (*Vav*^*iCre*^*Tet3*^*fx//fx*^) show normal frequency and numbers of myeloid, B lymphoid, and erythroid cells in BM but show a minor increase in the frequency of LSK HSPCs and a decrease in the frequency and absolute number of HSCs in BM. However, *Tet3* deficiency augmented the CHRC of HSPCs [92]. During hypoxia, *Tet3* is upregulated, thus promoting erythropoiesis, while during glucose deprivation stress, *Tet3* is upregulated to maintain systemic glucose homeostasis by upregulating glycolytic enzymes [93]. In human CD34^+^ HSPCs, *TET3* knockdown markedly impaired terminal erythroid differentiation, as reflected by increased apoptosis, the generation of bi/multi-nucleated polychromatic/orthochromatic erythroblasts, and impaired enucleation, in contrast to what is seen in *TET2* knockdown [91]. This suggests that TET2 and TET3 regulate the differentiation of erythroid progenitors at different stages.

Compound-knockout mice demonstrated that Tet3 has compensatory effects in preventing malignant transformation in *Tet2*^*−/−*^ mice among all lineages (Fig. 1 and Table 1). *Mx1*^*Cre*^*Tet2*^*fx/fx*^*Tet3*^*fx/fx*^ mice died of aggressive acute myeloid leukemia (AML) with a median survival of 10.7 weeks. *Mx1*^*Cre*^*Tet2*^*fx/*+^*Tet3*^*fx/fx*^ mice and *Mx1*^*Cre*^*Tet2*^*fx/fx*^*Tet3*^*fx/*+ ^mice developed AML at longer latencies, with a median survival of ∼27 weeks, suggesting a dose-dependent activity for Tet2 and Tet3 in AML development [20, 65]. The profound hypermethylation status of *Tet2* and *Tet3* double-knockout HSPCs and full-blown AML development in *Mx1*^*Cre*^*Tet2*^*fx/fx*^*Tet3*^*fx/fx*^ mice suggest the compensatory effects of Tet2 and Tet3 in the differentiation of early myeloid progenitors [20]. Although neither *Mb1*^*Cre*^*Tet2*^*fx/fx*^ nor *Mb1*^*Cre*^*Tet3*^*fx/fx*^ mice (deletion of *Tet2* or *Tet3* at the pro-B progenitor stage) displayed any striking B-cell abnormalities, *Mb1*^*Cre*^*Tet2*^*fx/fx*^*Tet3*^*fx/fx*^ specimens showed a block in B-cell development at the transition from the pro-B to pre-B cell stage due to focal DNA hypermethylation at enhancers that are enriched for consensus binding motifs of key B-lineage TFs such as Pu.1, Ebf1 and E2a [40, 64]. Consequently, percentages, and numbers of B-cells in BM were significantly reduced in these mice [40, 64]. *Mb1*^*Cre*^*Tet2*^*fx/fx*^*Tet3*^*fx/fx*^ mice develop fully penetrant B-cell lymphomas which resemble B-ALL by 5 to 6 months [40, 64]. Mechanistically, Tet2 and Tet3 regulate demethylation of the 3’ and distal Eκ enhancers of the *Igκ* locus, which is critical for BCR formation and pro-B-to-pre-B-cell differentiation. In mature B-cells in the spleen, Tet2 and Tet3 cooperatively control antibody production by regulating antibody class switch recombination (CSR) and shape the mutational landscape of GC B-cells [62]. Mechanistically, Tet2 and Tet3 are recruited to the enhancers of the *AID* gene by the TF Batf to control *AID* expression. Deletion of *Tet2* and *Tet3* in mature B-cells (*CD19*^*Cre*^*Tet2*^*fx/fx*^*Tet3*^*fx/fx*^mice) impairs the CSR of GC B-cells and inhibits plasma cell differentiation and leads to hyperactivation of B- and T-cells, CD86 upregulation and autoantibody production, and lupus-like disease in mice [63]. Interestingly, mice with *Tet2* and *Tet3* deletion in GC B-cells (*Cγ1*^*Cre*^*Tet2*^*fx/fx*^*Tet3*^*fx/fx*^ mice) do not develop such a phenotype, suggesting that Tet2/3 play such a role in naïve B cells upstream of GC cells. In T-cells, Tet2 and Tet3 collaboratively regulate the lineage commitment of CD4^+^ T cells and invariant NKT (iNKT) cells. Mice with T-cell-specific deletion of both *Tet2* and *Tet3* (*CD4*^*Cre*^*Tet2*^*fx/fx*^*Tet3*^*fx/fx*^) developed an aggressive PTCL-like syndrome that was apparent by 5 to 6 weeks of age, with all mice dying at ∼8 weeks. The malignant cells originated from iNKT cells in the thymus rather than from T follicular helper cells (Tfh) [17, 57]. In Treg cells, Tet2 and Tet3 control DNA demethylation of the *FoxP3 CNS2* enhancer and the stability of FoxP3 expression. Loss of *Tet2* and *Tet3* converts Treg cells into Tfh/Th17 phenotypes due to the reduction of *FoxP3* expression. *FoxP3*^*Cre*^*Tet2*^*fx/fx*^*Tet3*^*fx/fx*^mice develop autoimmune/inflammatory disease [66].

Taken together, all 3 Tets are involved in regulating the fate determination of HSPCs at multiple differentiation steps by mediating stepwise changes in the epigenetic landscape and transcriptional networks. Among the 3 Tets, Tet2 is the major player here. Deletion of *Tet2* causes DNA hypermethylation and reduced 5hmC in the enhancers of lineage-specific genes, disrupting lineage commitment and differentiation in the corresponding differentiation stages. Tet2 plays such roles primarily through catalytic activity-mediated site-specific DNA demethylation. Tet2 also has non-catalytic activity which is involved in the regulation of many hematopoietic cell behaviors such as HSC self-renewal, mast cell proliferation and monocyte/macrophage cytokine production. However, due to the functional compensation of Tet1 and/or Tet3, the phenotype of young *Tet2*^*−/−*^ mice is relatively mild [20, 40, 64, 67, 94]. The disease phenotype in aged *Tet2*^*−/−*^ mice is mainly induced by inflammatory cytokines and accumulated additional mutations. Tet3 compensates for the function of Tet2 in almost all differentiation stages studied. However, Tet1 compensates for the function of Tet2 in B-cell and T-cell lineages but antagonizes the function of Tet2 in HSPCs and myeloid lineages. All of the Tets play their roles by collaborating with lineage-specific pioneer TFs and inducing lineage-specific gene expression.

### Role of TET dioxygenases in the regulation of malignant hematopoiesis

#### Somatic mutations of TETs in human hematologic malignancies

Mutations in *TET1* occur at a much lower frequency than *TET2* in hematopoietic malignancies. Mutant *TET1* was first identified as a fusion partner of the *MLL* gene in patients with AML carrying a t(10,11)(q22;q23) mutation; such translocations are, however, very rare [95, 96]. In this fusion protein, the TET1 fragment lacks catalytic activity. Thus, it is believed that the MLL-TET1 fusion protein induces the development of AML by recruiting TET1 partner proteins to MLL target genes [97]. The mutation or downregulation of *TET1* is frequently found in patients with non-Hodgkin B-cell lymphoma, including DLBCL and follicular lymphoma [44, 98, 99]. In addition, *TET1* is also mutated in 12–15% of T-cell acute lymphoblastic leukemia and in 1–5% of AML patients [100, 101].

*TET2*^*MT*^ has frequently been found in human patients with myeloid malignancies [102–104] such as 7.3%-23% of AML, 18%-33% of MDS, 46% of MDS/myeloproliferative neoplasms (MPNs), 13–20% of MPNs, 22%-56% of CMML, and 20.3–29% of systemic mastocytosis, as well as subtypes of mature B/T-cell malignancies [36, 43, 61, 105–108] including 4% of mantle cell lymphomas, 2–10% of DLBCLs, 42–89% of angioimmunoblastic T-cell lymphomas (AITL), 28–48.5% of peripheral T-cell lymphomas (PCTL-NOS), ∼2.3% of CLL, and 5% of multiple myelomas (MM) [109]. Germline TET2 loss of function causes childhood immunodeficiency and lymphoma [110]. In addition, *TET2*is the second-most frequently mutated gene (11–15%) in ARCH [22, 23]. While many mutations lead to loss of the entire protein (47% frame-shifts and 34% nonsense), 19% are missense mutations involving either the catalytic or non-catalytic domains, which lead to either loss of catalytic activity or disruption of interactions with key partners [33].

*TET3* mutations are the least common among the three TET genes in hematopoietic malignancies. Inactivating *TET3* mutations are very rarely identified in peripheral T-cell lymphomas (PTCLs) [107] and CLL [111].

#### The expression and functions of TETs in human hematologic malignancies

**Fig. 2:**
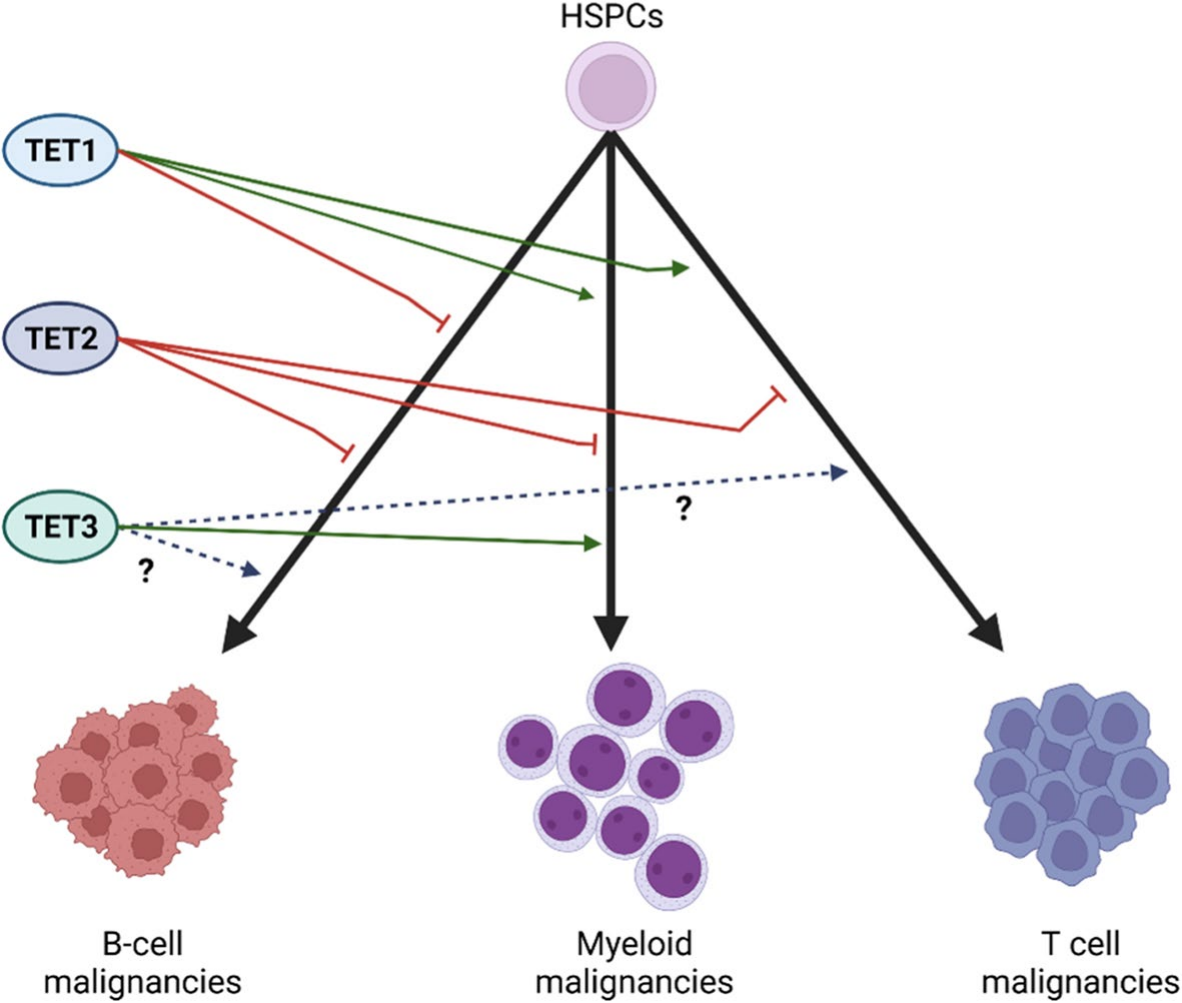
The roles of TETs in the pathogenesis of human hematopoietic malignancies. Studies of human hematopoietic malignancies suggested that TET2 is a tumor suppressor for almost all types of hematopoietic malignancies, while TET1 is a tumor suppressor for B-cell malignancies but a tumor promotor for myeloid or T-cell malignancies. TET3 is required for the survival and proliferation of myeloid malignancies. However, its role in T- and B-cell malignancies has yet to be determined. (Created with BioRender.com)

TET2 has a pleiotropic role in hematopoiesis [112]. TET2 is a tumor-suppressor protein in all types of hematopoietic malignancies as determined by inactivating mutations, described above. In ALL, although *TET2* mutations are not reported, *TET2* is transcriptionally repressed or silenced in 71% and 17% of T-ALL, respectively, and is often associated with hypermethylation of the *TET2*gene’s promoter [113]. Reduced 5hmC in T-ALL caused by reduced *TET2* expression is associated with more aggressive malignancies with worse prognoses. In addition, *TET2* expression in some leukemic cells is repressed by microRNAs. There are more than 30 miRNAs that inhibit *TET2* expression, including miR-22 [114]. miR-22 promotes HSC self-renewal and leukemic transformation by repressing TET2 [115]. However, both tumor-promoting and tumor-repressive functions of TET1 and TET3 have been reported. Such tumor-promoting and repressive functions of TET1 and TET3 are most likely cancer type-specific (Fig. 2).

The tumor-promoting role of TET1 has been reported in both myeloid and T-cell malignancies [26, 45, 46]. Aberrant overexpression of *TET1* has been observed in AML with MLL-fusion proteins. Knockout/knockdown of *Tet1* suppresses AML development in *MLL-AF9*-transduced murine AML models [95]. Tet1 is involved in MLL-AML development by promoting the expression of oncogenic target genes such as *Hoxa9*, *Meis1*, and *Pbx3* [95]. However, a recent study demonstrated that Tet1 is not required for AML pathogenesis in an *MLL-ENL* mouse model [116]. Such a discrepancy might be explained by a difference in the *MLL-*fusion gene or the techniques used for fusion gene induction. In cytogenetically normal AML patients, higher TET1 expression is correlated with lower overall survival [117]. The aberrantly high expression of the TET1 protein regulates the expression of critical oncogenic pathways in AML cells. Targeted inhibition of the STAT/TET1 axis has been proposed as a therapeutic strategy for TET1 high-expressing AML [95, 118]. In addition, TET-1s is overexpressed in many other cancer types including AML [44, 47, 117, 118]. Nevertheless, the role of TET1s in leukemogenesis will need to be further determined in the future. In the majority of human T-ALL cells, TET1 protein is upregulated by PARPs via PARylation-mediated DNA/histone modification of the *TET1* gene promotor and TET1 protein stability [44, 119]. High levels of TET1 positively regulate oncogene expression (such as for *NOTCH3*), safeguarding genomic integrity, and thereby promoting T-ALL development by maintaining global 5hmC [120]. The PARP inhibitor Olaparib abrogates TET1 expression, induces the loss of 5hmC, and antagonizes the growth of T-ALL cells [120]. Another study suggested that overexpression of TET1 and down-regulation of TET2 are mediated by MYC in T-ALL cells. TET1 and TET2 are functionally opposed to T-ALL cell growth by regulating distinct 5hmC patterns in the genome [121]. The tumor-suppressive role of TET1 in B-cell malignancies has been well-documented [44]. In addition to inactivating mutations, *TET1* downregulation is commonly detected in B-cell malignancies including B-ALL, B-cell lymphoma, and MM [44, 98, 99, 122]. Both *TET1 and TET2* are often concomitantly downregulated in B-ALL [41, 44]. The decrease of *TET1* expression in these malignancies is associated with phenotypic hypermethylation of enhancers [44, 98, 99, 122]. Downregulation of *TET1* could be regulated by HMGA2 and PRC2-mediated epigenetic promotor methylation [123, 124], miRNA-mediated posttranscriptional repression [125], or calpain-mediated degradation of TET1 protein [126]. A CpG island has been identified in the *TET1* promoter and in the exon 1 region, which is commonly methylated in B-cell malignancies including both Hodgkin’s and non-Hodgkin’s lymphomas, NK/T-cell lymphomas, and MM [44, 127, 128]. TET1 and TET2 activities are also downregulated in several types of cancer by XPO1-mediated nuclear exportation, which can be restored by the XPO1 inhibitor leptomycin B (LMB) [129]. The tumor repressive activity of TET1 in the B-lymphocyte lineage has been verified in *Tet1*^*−/−*^*Tet2*^*−/−*^mice [41, 44].

The role of TET3 in the pathogenesis of AML is reported differently in various studies. Early studies reported that TET3 is down-regulated in aged HSPCs, peripheral blood T-cells, and human AML samples [130]. These studies suggested that TET3 is a repressor of AML. Such AML repressive activity for TET3 has been verified in two recent studies using *Tet2−/−Tet3−/−* mice [59, 87] (see above section). However, some other studies reported that TET3 expression is significantly increased in some MDS patients [38] and a majority of AML patients, specifically within leukemic stem cell (LSC) populations [39, 45, 131]. In MDS patients, downregulation of TET2 and reduction of 5hmC levels are commonly detected irrespective of TET2 mutations, while TET3 is upregulated, this being inversely correlated with TET2 expression, likely due to a feedback mechanism. Elevated TET3 levels were positively associated with good outcomes for TET2-mutant MDS [38]. In AML cells, TET3 expression was positively correlated with tumor suppressor gene expression, including CDKN2B, ZIC2, and miR-196a, and negatively correlated with oncogenes such as PAX2 and IL-2RA in AML specimens. In addition, TET3 regulates the expression of genes involved in the early myeloid progenitor program, critical glucose metabolic pathways, and the STAT5A signaling pathway; it does so by maintaining 5hmC epigenetic marks [45]. Furthermore, TET3 expression is negatively associated with overall survival and disease-free survival in AML patients [39, 45]. A functional study demonstrated that overexpression of either TET3 or TET-3s promotes AML progression by epigenetically regulating glucose metabolism and LSC-associated pathways [45]. TET3 depletion causes a dramatic impact in 5hmC marks, apoptosis, and growth of AML cells in vitro and in vivo. In addition, TET3 depletion also renders AML cells highly sensitive to the combination of 2-deoxy-D-glucose and STAT5 inhibitor treatment. A TET-selective small molecule inhibitor, TETi76, decreases 5hmC and restricts clonal outgrowth of TET2^MT^ HSPCs in vitro and in vivo. These results suggest that TET inhibitors may constitute a new class of targeted agents for TET2^MT^ neoplasia [131]. Nevertheless, the apparent opposing roles played by TET3 in AML, as demonstrated by simultaneous deletion of Tet2 and Tet3 in HSPCs in mice and shRNA knockdown or TET inhibitor treatment in human AML cells, are not explained by differences between the human and mouse diseases because TET3 inhibition also represses the growth of murine AML cells. It is also not due to the expression of TET3 isoforms because it was found that both TET3 and TET3s have similar growth-promoting activities in AML cells. It is most likely that such contradictory roles are related to the disease stage or time period of TET3 inhibition. It is also possible that inhibition of TET3 might only be a selective subset of TET2^MT^ HSPCs. The remaining TET2^MT^ HSPCs undergo additional signaling changes or genetic abnormalities when TET3 is inhibited, causing AML development. The roles of TET3 in the pathogenesis of B- and T-cell malignancies have not yet been adequately studied.

### Concomitant mutations in *TET2*^*MT*^ hematopoietic malignancies

Somatic *TET2*^*MT*^ can be detected in ARCH. This suggests that *TET2*^*MT*^ alone creates a leukemogenic predisposition by altering the 5hmC/5mC ratio on the active enhancers of its target genes, inhibiting the access of key TFs for target gene expression and DNA stability [18]. Additional mutations are required for full malignant transformation. The long-term latency of disease development in *Tet2*^*−/−*^ mice suggests that *Tet2* deficiency predisposes to, but depends on, additional oncogenic hits to induce the development of full-blown hematological malignancies [13, 16, 18, 35, 43, 60, 61, 106, 118] (Table 2).

**Table 2:** Collaboration of other leukemic oncogenes with Tet2^MT^ in malignant hematopoietic development in mouse models

Mouse lines	HSCs and MPPs	Lifetime	Hematopoietic diseases
*Vav*^*cre*^*Tet2*^fx/fx^*Jak2*^*V617F*^ mice [132] *TET2*^*trap/*+^*Jak2*^*V617F*^ mice [133]	Enhanced competitive advantage to Jak2^V617F^-mutant HSCs *TET2* loss prevents the exhaustion of *JAK2*^*V617F*^ HSCs Sustain MPNs over long periods of time	1–6 months	Accelerated MPNs
*Cre*^*ERT*^*Ezh2*^fx/fx^*Tet2*^*trap/trap*^ mice [134]	Enhanced repopulating capacity of HSCs and extramedullary hematopoiesis	10 months	Enhanced pathogenesis of MDS/MPN, MDS
*Mx1-cre Asxl1*^fx/fx^*Tet2*^fx/fx^ mice [135]	*Tet2* deletion restores self-renewal of *Asxl1*-deficient HSCs	6–7 months	Developed MDS phenotype with hastened death
*Mcpt5*^*Cre*^*Tet2*^fx/fx^*Kit*^*D816V* ^ [136, 137]	*Tet2* deletion increases proliferation and impairs differentiation of BM mast cells *Tet2* deletion in BM mast cells induces *c-MYC* upregulation via PI3K activation. Block in the differentiation of *KIT D814V* positive BMMCs	9 months	More aggressive forms of mastocytosis
*Vav*^*cre*^*Tet2*^fx/fx^*Flt3*^*ITD*^ mice [138] *Tet2*^−/−^*Flt3*^*ITD*^ mice [139]	*Flt3*^ITD^ and *Tet2* loss cooperatively remodeled DNA methylation and gene expression to an extent not seen with either mutant allele alone, including at the *Gata2* locus Expansion of myeloid cell compartment, and defects in maturation Alters the BM microenvironment and produces more pro-inflammatory cytokines including IL-5, IL-6, CXCL5, MIP-1A, MIP-1B, MIP-2, TNFα, IL-13, and IL-15	9–12 months 5 months	Develop AML refractory to standard AML chemotherapy and FLT3-targeted therapy AML
*AML1-ETO/Cre*^*ERT*^*Tet2*^fx/fx^, an infection and transplantation model [18].	Hypermethylation of enhancer elements results in lowered gene expression	6 months	Greatly accelerated onset of AML
*DNMT3A*^*R882H*^*Tet2*^*−/−*^ mice, an infection and transplantation model [140]	Accumulation of mutant HSPCs with impaired differentiation capability	within 6 months	AITL-like, AML-like, and T-ALL-like diseases in first transplantation recipients and a majority of AITL-like diseases in secondary recipients
*Mx1*^*cre*^*Dnmt3a*^*fx/fx*^*Tet2*^*−/−*^ mice [141]	Marked increases in LSK HSPCs Synergistic dysregulation of HSC- and RBC-associated genes *Klf1* and *Epor* erythroid genes promote mutant HSPC self-renewal	5 months	BM transplantation recipient mice die of multiple hematologic abnormalities 1. ~ 10% BM failure 2.50% T-cell thymic lymphoma 3.B220^+^CD19^−^ salivary gland infiltration, mature B-cell lymphoma in primary mice and develop B-ALL in recipients
*Vav*^*cre*^*Ncstn*^*fx/fx*^*Tet2*^*fx/fx*^ [142]	Enlargement of the GMP compartment due to differentiation defects	6 months	Die of AML-like diseases
*Mx1*^*Cre*^*Tet2*^fx/fx^*Nras*^G12D^ mice [143, 144]	Expansion of HSCs and MPPs. Increased response to cytokine stimulation. Enhanced HSC competitiveness and self-renewal	9–12 months	Accelerated, transplantable CMML disease AML [143]
*Vav-*^*iCre*+^*Pu.1*^*URΕ∆/*+^*Tet2*^*fx/fx*^ *Vav-*^*iCre*+^*Pu.1*^*URΕ∆/*+^*Tet2*^*fx/*+^ (with 30% reduction of Pu.1) [72]	Age-related reduction of Pu.1 expression. Increased methylation in Pu.1 binding motifs	10–20 months	Develop AML during aging with median survival 623 and 290 days respectively
*IDH2*^R140Q^*Flt3*^ITD^ mice [145]	*IDH2*^R140Q−t^ induces a block of erythroid differentiation in KSL cells	7 months	AML with T-cell markers
*Hmga2* expression in *Tet2*^*−/−*^ mice [146]	Hmga promotes Igf2bp2 expression and impairs differentiation of *Tet2*^*−/−*^ myeloid cells		Progressive MDS and AML
*ERT*^*Cre*^*Tet2*^fx/fx^*Bcor*^*ΔE9−10/y*^ mice [147]		3–6 months	Progressive MDS
*Mx1-Cre Sf3b*^*1K700E/*+^ *Tet2*^−/−^ mice [148]		12 weeks	Earlier onset and more severe MDS
**T-cells**			
*Vav*^*cre*^*Idh2*^*R172K*^ mice [149]	Slight increase in 2-HG levels in ICOS^+^ T_fh_ cells	Only studied for 3–7 month olds	Impairs lymphocyte development. CD4^+^ and CD8^+^ naive T-cells were decreased, while CD8^+^ central memory cells were increased
*CD4-RhoA*^*G17V*^ TG mice [58]	Relatively increased TFH-cell populations are accompanied by markedly reduced naive T-cells		Autoimmunity due to CD4^+^ Th17 cell infiltration
*RhoA*^*G17V*^ transduction of *Tet2*^−/−^ *T* cells [150]	Increased Ki67^+^CD4^+^ T-cells, CD4^+^CD44^ +^ T-cells and CD4^+^CXCR5^+^Bcl-6^+^Tfh Reduction in Treg and FAS^ + ^GL-7^+^ GC B- cells Partial AITL gene signature Increased cytokine production, such as IL-6 and INFγ	5 months	Inflammatory diseases or aggressive cancer (PTCL-Tfh) developed
*Cd4*^*CreER*T2^*Tet2*^fx/fx^*Rhoa*^G17V^ mice [151]	Tfh (CD4^+^CXCR5^+^PD1^+^, ICOS^+^, Bcl-6^+^) Tfh gene signature AITL gene signature	6 months	Aggressive AITL-like lymphomas
*Vav*^*Cre*^*Tet2*^fx/fx^*Cd4*-*Rhoa*^G17V^ (transgene) [58] *Vav*^*Cre*^*Tet2*^fx/fx^*Cd4*-*Rhoa*^G17V^ *OT-II* mice	Tfh (CD4^+^CXCR5^+^PD1^+^Bcl-6^+^)	7 months	PTCL and developed autoimmune syndromes with Tfh cell expansion and autoantibody generation Mice bearing an OT-II T-cell receptor transgene developed AITL-like lymphomas

In patients with *TET2*^*MT*^ hematopoietic malignancies, *TET2*^*MT*^ is not only detected in malignant cells but also in CD34^+^ HSPCs and other lineages of non-neoplastic blood cells in ~ 40% of cases, suggesting that *TET2*^*MT*^ is the first hit (*i.e.*, ancestral dominant clone) occurring before leukemia or lymphoma develops [35, 36, 103]. However, *TET2*^*MT*^ in the remaining ~ 60% of cases are later hits (*i.e.*, subclonal events). In the cases with *TET2*^*MT*^ as first hits, the most common second mutation is another *TET2* lesion, followed by *SRSF2, ASXL1, DNMT3A,* and *SF3B1* mutations [33]. The loss of the second allele of *TET2* suggests clonal selection for a complete loss of *TET2* for the clonal evolution and malignant transformation. Consistently, in *TET2*^*MT*^ hematopoietic malignancies, ~ 43% of cases are biallelic [33]. In cases with *TET2*^*MT*^ as later hits, the dominant antecedent clone is defined by the presence of *SRSF2, EZH2, ASXL1, TP53, U2AF1, DNMT3A,* or *CEBPA* mutations. In many cases, the order of *TET2*^*MT*^ with other co-occurring mutations is not only associated with disease identity but is also related to patient outcomes. For example, in MPN patients, *TET2* mutations are more common in patients with myelofibrosis than those with essential thrombocythemia [152]. In *JAK2*^*V617F*^ MPN patients, *TET2* mutations can either present as the first hit or a second hit [37]. Interestingly, “*JAK2*-first” patients presented with significantly worse overall survival compared to “*TET2*-first” patients [37].

#### Concomitant mutations in TET2^MT^ myeloid malignancies

**Fig. 3:**
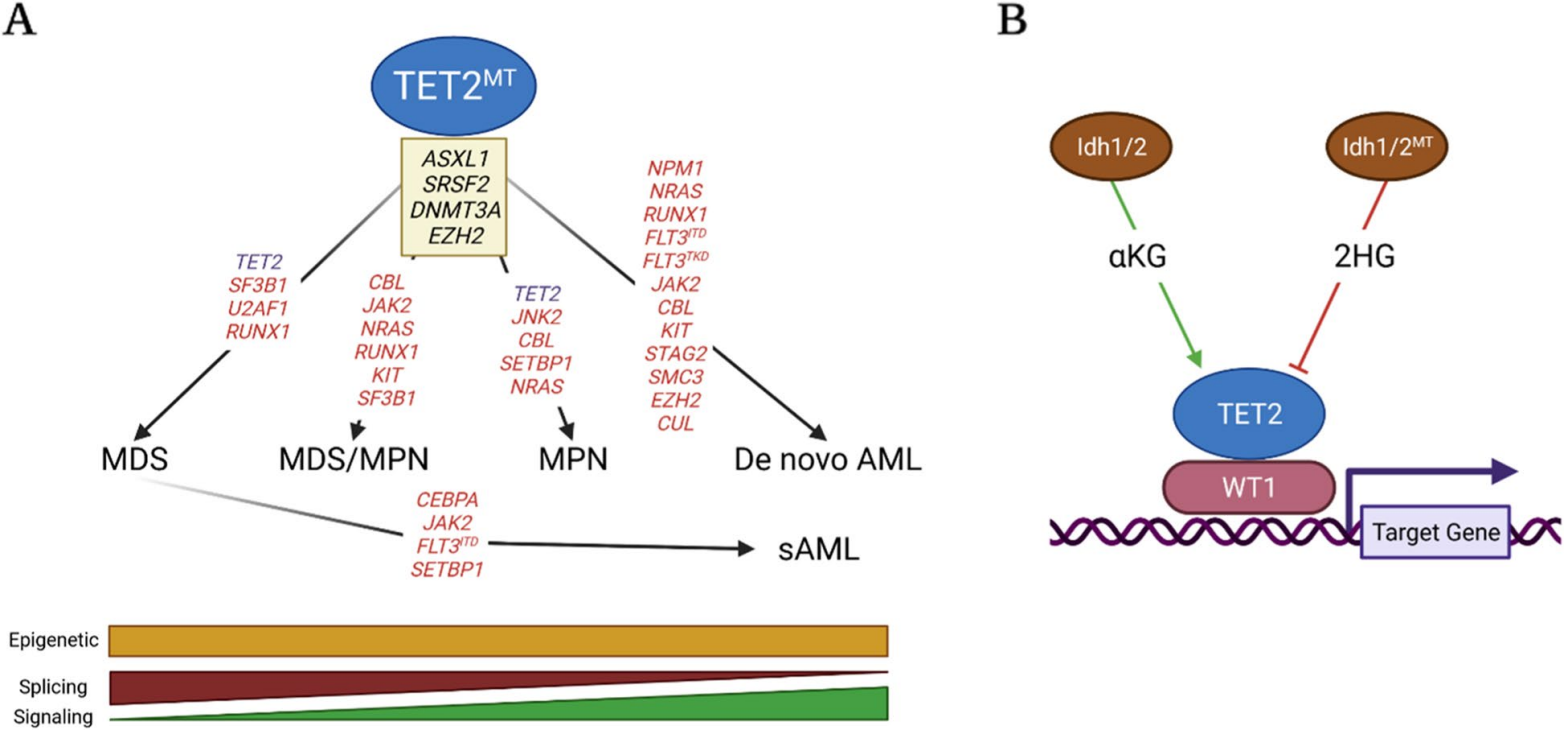
Concurrent genetic mutations of *TET2*^*MT*^ human myeloid malignancies. **A**. Mutations of *ASXL1, SRSF2, DNMT3*A, and *EZH2* concur in all types of myeloid malignancies. Second allele mutations of *TET2* are commonly detected in MDS and MPNs but not in de novo AML. In addition, mutations in splicing factors such as SF3B1 and U2AF1 are commonly detected in MDS, while mutations of signaling molecules are commonly detected in MPN and AML patients. **B** Idh1/2 regulate the production of α-KG and promote TET2 activity, whereas mutant Idh1/2 regulate the production of 2HG and repress TET2 activity. TET2 regulates the differentiation of myeloid progenitors primarily by interacting with WT1 for DNA binding. Mutations of *Idh1/2* and *WT1* are exclusive in *TET2*^*MT*^ myeloid malignancies. (Created with BioRender.com)

Among these concurrent mutations, the frequencies of some mutations in *TET2*^*MT*^ cases are higher than in *TET2* WT cases (*TET2*^*WT*^), indicating that such mutations might preferentially promote *TET2*^*MT*^ malignancies, while the frequencies of some other mutations in *TET2*^*MT*^ cases are comparable to *TET2*^*WT*^ cases, suggesting that these mutations promote malignant transformation without selection. For example, biallelic *TET2*^*MT*^ are commonly detected in MDS and secondary AML patients (sAML), suggesting that the disease progresses from *TET2*^*MT*^ ancestral clones. In addition, the frequencies of *SF3B1, ASXL1, SRSF2, RUNX1, DNMT3A,* and *EZH2* gene mutations are significantly higher in *TET2*^*MT*^ MDS cases compared to *TET2*^*WT*^ MDS cases, while the frequencies of *SRSF2*, *ASXL1*, *RUNX1*, *CEBPA*, *DNMT3A, JAK2*, *FLT3*^*ITD*^*,* and *SETBP1* gene mutations are significantly higher in *TET2*^*MT*^ sAML cases compared to *TET2*^*WT*^* s*AML cases. This suggests a collaborative role for *SRSF2*, *ASXL1*, *RUNX1*, *CEBPA*, *DNMT3A,* and *TP53* mutations with *TET2*^*MT*^ in MDS development and sAML progression. *JAK2*, *FLT3*^*ITD*^, *N-RAS,* and *SETBP1* mutations are later events that are critical for MDS to transform to sAML [153]. Compared to *TET2*^*WT*^ MDS/MPN and MPNs, a higher frequency of *SRSF2, ASXL1, RUNX1, CBL, JAK2, N-RAS,* and *SF3B1* mutations were observed in *TET2*^*MT*^ MDS/NPM, whereas a higher frequency of *JNK2, ASXL1, SRSF2, TET2, CBL, SETBP1, N-RAS,* and *EZH2* mutations were seen in *TET2*^*MT*^ MPNs. In de novo AML, higher frequencies of *NPM1*, *DNMT3A, CEBPA*, *ZRSR2, ASXL1,* and *N-RAS* mutations were observed in *TET2*^*MT*^ cases compared to *TET2*^*WT*^ cases, while *FLT3-ITD*, *FLT3-TKD*, *JAK2*, *RUNX1*, *CEBPA*, *CBL,* *KIT, SMC3, CBL, EZH2,* and *CUL* mutations are comparable between *TET2*^*MT*^ and *TET2*^*WT*^ cases [154, 155]. Therefore, *ASXL1*, *SRSF2*, *DNMT3A,* and *EZH2* mutations commonly concur in all types of *TET2*^*MT*^ myeloid malignancies, while splicing factor mutations (*e.g.*, *SF3B1* and *SRSF2*) more commonly occur in MDS and MDS/MPN cases, JAK2*, CBL,* and *N-RAS* kinase mutations more commonly occur in MPN and MDS/MPN cases, *NPM1, FLT3-ITD, JAK2, CBL, c-KIT,* and isolator mutations are more common in AML (Fig. 3A).

Some genetic mutations present at much lower frequencies or are even exclusive of *TET2*^*MT*^, suggesting that these mutations might either be functionally redundant with *TET2*^*MT*^ or toxic to *TET2*^*MT*^ cells. For example, *TET2*^*MT*^ is mutually exclusive to *WT1* and *IDH1/2* mutations in all types of myeloid malignancies (Fig. 3B) [156–158]. WT1 physically interacts with and recruits TET2 to its target genes [156]. Mutations in *WT1* induce a similar effect to that of *TET2*^*MT*^, suggesting a common pathway may exist for TET2 and WT1 [157]. Mutant IDH1 or IDH2 convert isocitrate to 2-hydroxyglutarate (2HG) instead of α-ketoglutarate (α-KG). 2HG inhibits TET2 activity by competing with α-KG. Thus, *IDH1/2* mutations can mimic the effect of *TET2*mutations, leading to a similar transcriptomic profile [159]. A recent study suggested that 2HG is synthetically lethal to *TET2*^*MT*^in myeloid tumors [131]. Two recent studies suggested that TET3 is required for the growth of human *TET2*^*MT*^AML cells [45, 131], suggesting that *IDH1/2* mutations might selectively repress *TET2*^*MT*^ myeloid malignancies by 2HG-mediated inhibition of TET3. Interestingly, *IDH1/2* and *TET2* mutations commonly co-occur in AITL, which will be discussed in the following section. In DLBCL patients, *TET2* and *CREBBP* mutations are mutually exclusive, suggesting that TET2 and CREBBP cooperate to regulate cell differentiation and cell-cycle exit and to prevent lymphomagenesis in GC B-cells [61]. Thus, it is not surprising that *TET2* and *CREBBP* mutations show a similar impact on the transcriptional profile of the affected cells. Moreover, *TET2*^*MT*^ was found to be coincidental with rarely occurring somatic mutations such as *MPL*^*W515L*^ and *PML-RARα* within the BM hematopoietic cells derived from MDS patients [153].

#### Concomitant mutations in TET2^MT^ PTCL

**Fig. 4:**
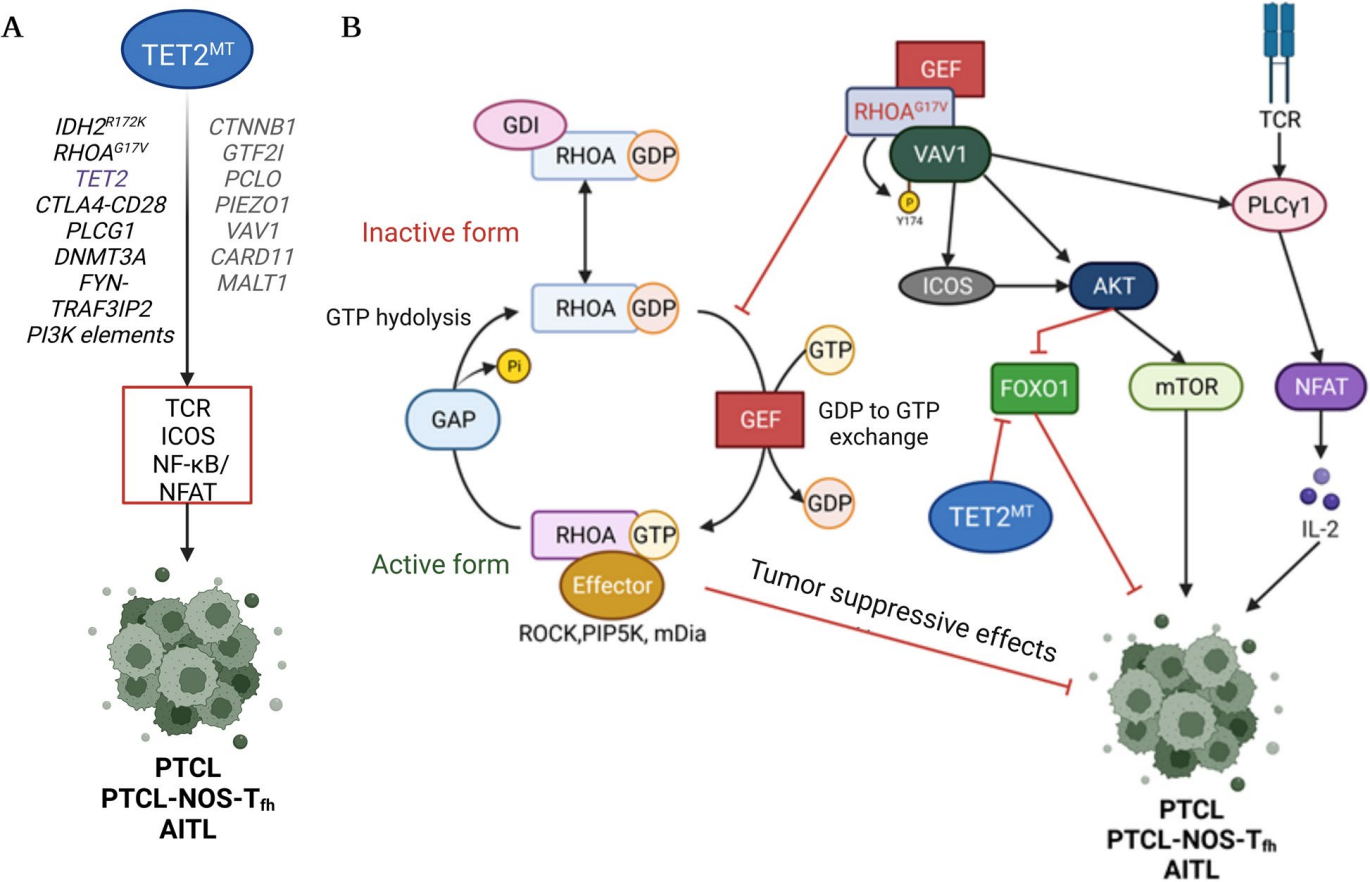
Concurrent genetic mutations of TET2^MT^ PTCL. **A**. RHOA^G17V^ mutation and mutations of key components of TCR and ICOS signaling pathways such as *CD28*, *PLCG1,* and *VAV1* commonly co-occur in *TET*^*MT*^ PTCL. Consequently, TCR and ICOS signaling are activated in *TET2*^*MT*^ PTCL, determining the Tfh phenotype. In addition, second *TET2* mutations are commonly detected in *TET2*^*MT*^ PTCL. Moreover, IDH2^R172K^ mutation also commonly co-occurs in *TET2*^*MT*^ PTCL. **B**. The molecular mechanism of RHOA^G17V^ mutation in the pathogenesis of PTCL in collaboration with TET2^MT^. RHOA^G17V^ mutation antagonizes the normal function of RHOA and activates ICOS-AKT-mTOR and PLCγ1-NFAT signaling by stimulating the activation of VAV1. TET2^MT^ collaborates with RHOA^G17V^ mutation in the regulation of FoxO1 activity in T-cells. (Created with BioRender.com)

As is the case with MDS, a majority of PTCL patients with *TET2*^*MT*^ harbored more than one *TET2* mutation, presenting as either bi-clonal/oligoclonal T- cells or bi-allelic mutations in a single clone, suggesting a clonal evolutionary mechanism in *TET2*^*MT*^ T-cell clones [106]. Malignant cells from *TET2*^*MT*^ PTCL patients express TFH markers (such as CD10, CXCL13, ICOS, PD-1, and BCL6) and TFH gene profiling, suggesting they are derived from TFH cells. Almost all *TET2*^*MT*^ PTCLs are subclassified into either AITL or PTCL-NOS (TFH) [109, 136]. Interestingly, as distinct from myeloid malignancies, IDH2^R172^ mutations commonly co-occur with *TET2*^*MT*^ in PTCL. The IDH2^R172K^ mutation is observed in 20–45% of AITL cases and 7.5% of PTCL-NOS patients (Fig. 4A) [137]. *TET2*^*MT*^ are present in 60 ~ 100% of IDH2^R172K^-mutant AITLs [106, 137]. A study suggests that, compared to other mutant *IDH2*, IDH2^R172K^ might generate a lower concentration of 2HG, which impairs lymphocyte development but is less toxic to *TET2*^*MT*^ T-cells [160]. In addition to TETs, 2HG also inhibits more than 60 other α-KG-dependent dioxygenases including JmjC domain-containing histone demethylases (KDMs) and ATM, which are involved in multiple cellular functions including epigenetic regulation, DNA repair, HIF1α regulation, and collagen maturation [160]. Therefore, there is a possibility that 2HG produced by IDH2^R172K^ promotes *TET2*^*MT*^ T-cells by inhibition of other α-KG-dependent dioxygenases [160].

The most commonly concurring mutation in *TET2*^*MT*^ PTCL is the RHOA^G17V^ mutation. RHOA^G17V^ is detected in ~ 60–70% of *TET2*^*MT*^ AITL patients (Fig. 4A). RHOA^G17V^ functions as a dominant-negative mutation that competes with WT RHOA and probably with other RHOA family members as well, for guanine nucleotide-binding. Thus, RHOA^G17V^ represses canonical RHOA signaling. In addition, RHOA^G17V^ also presents with some additional functions. For example, Fujisawa et al*.* demonstrated that RHOA^G17V^ gains the ability to bind to VAV1 and promotes VAV1-PLCγ1-NFAT signaling downstream of TCR. Other studies have found that RHOA^G17V^ enhances TFH lineage specification by the activation of mTOR signaling, probably through up-regulation of ICOS [106, 161]. These studies suggest that activation of TCR and ICOS signaling might promote AITL development in combination with *TET2*^*MT*^ (Fig. 4B) [162]. Consistent with this conclusion, the concurrence of activating mutations in the key components of TCR and ICOS signaling have been identified in *TET2*^*MT*^ AITL and PCTL-NOS-THF, including 9% *CD28*, 38% *CTLA4-CD28* fusions, 14–23% *FYN-TRAF3IP2* fusions, 11% *VAV1,* 14% *PLCG,* 7% PI3K elements, 6% *CTNNB1*, 6% *GTF2I*, 23% *PCLO* and 17% *PIEZO1* (Fig. 4A) [146, 150]. In addition, mutations in the JAK-STAT pathway, such as mutations in *JAK1*, *STAT3,* and *STAT5*, coincide with *TET2*^*MT*^in PTCL patients [162, 163]. CD8^+^ PTCLs are characterized by concurring *DNMT3A* and *TET2*mutations [164].

#### Clonally heterogenic architecture of hematopoietic malignancies

Next-generation DNA/RNA sequence assays showed a dynamic accumulation of driver mutations during the development and progression of hematopoietic malignancies. It was suggested that the average number of driver mutations is increased from 1 in ARCH to 3 in MDS and > 5 in AML [22, 23, 165]. Single-cell approaches, including single-cell DNA-seq, single-cell RNA-seq, and single-cell proteomics, demonstrated a clonal heterogeneity in the architecture of these hematopoietic malignancies due to both linear and branching clonal evolutionary processes in disease development. For example, in AML samples, based on mutational history, pre-leukemic clones, MDS clones, and multiple AML clones can be detected in the same patient. Although all clones in the same patients share the same founder mutation, such as *TET2*^*MT*^, the subsequent mutations are not the same among them [166, 167]. Because the therapeutic responses of different clones are not all the same, some clones are completely eliminated during treatment and only AML cells from certain clones are sustained, which eventually leads to disease relapse. In many patients, new mutations are acquired in some clones which provide a growth advantage leading to disease relapse. Moreover, the clonal architectures in different patients are different. Thus, the dynamic and heterogenic clonal evolutionary processes occurring in these malignancies make the diseases difficult to treat [165]. Therefore, understanding the unique biology of each clone by elucidating how the concurrent mutations collaborate in the induction, development, and progression of the disease will suggest novel combinatory strategies for eventually curing these fatal diseases. Genetically modified animal models will continue to provide useful platforms to examine and address such issues.

#### Oncogenic collaboration of mutations with Tet2^MT^ in animal models

The potential oncogenic cooperation effect of commonly-concurring mutant genes with the inactivation of *Tet2* has been evaluated in numerous animal models. Consistent with *TET2*^*MT*^ in human hematopoietic malignancies, *Tet2* deficiency in mice induces a “poised” state in pre-leukemic HSPCs through altered gene expression. *Tet2*^*−/−*^ mice develop CMML-like disease and T/B-cell malignancies with long-term latencies after acquiring additional mutations. In the tumors which develop in *Tet2*^−/−^ mice, numerous mutations, including *Apc*, *Nf1*, *Flt3*, *Cbl*, *Notch1*, and *Mll2*, have also been detected in human hematological malignancies [42]. These accumulated mutations not only drive the aberrant proliferation and survival of the *Tet2*^−/−^ cells for malignant development but also drive the lineage commitment and differentiation of the mutant cells to determine the identity of the malignant cells.

**Fig. 5:**
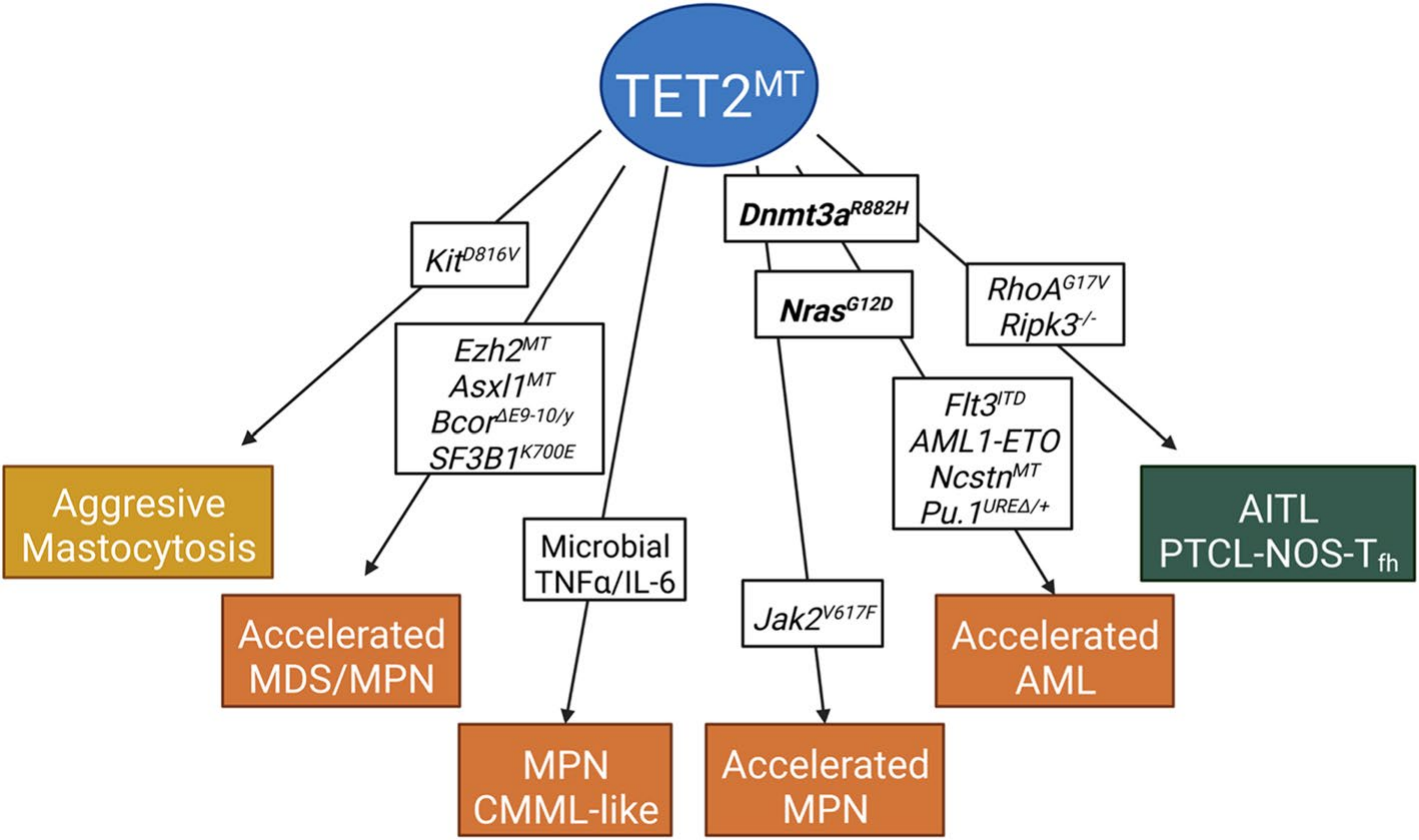
Oncogenic collaboration of mutations with TET2^MT^ in animal models. *Tet2*^MT^ mice develop MPNs, AITL-like, AML-like, or T-ALL-like diseases when combined with Dnmt3A^R882H^ mutation or *Dnmt3A *deletion, and accelerated MPN or AML when combined with N-ras^G12D^ mutation. However, *Tet2*^*MT* ^mice develop AITL, AML, MPN, MDS/MPN, or mastocytosis when combined with RhoA^G17V^, *Flt3*^ITD^*/AML1-ETO/Ncstn*^MT^, Jak2^V617F^, *Ezh2*^MT^*/Asxl1*^MT^*/Bcor*^MT^ or Kit^D816V^, respectively. All these mutant phenotypes resemble the disease phenotypes of patients having the same combinations of mutations. (Created with BioRender.com)

Compound-mutant mouse experiments demonstrated that combinations of two concurrent mutant genes in mice induce hematopoietic malignancies that largely resemble similar diseases in patients (Fig. 5 and Table 2). For example, the concurrence of *DNMT3A* and *TET2* mutations was detected in patients with almost all types of hematopoietic malignancies [168]. Consistent with this, mice with compound *Dnmt3a* and *Tet2* mutations (including *Dnmt3a*^*−/−*^*Tet2*^*−/−*^ or *DnMt3a*^*R882H*^*Tet2*^*−/−*^) developed multiple types of hematologic malignancies including AITL, AML, and T-ALL. Mechanistically, it was found that loss of *Dnmt3a* maintains an HSC transcriptional program, and loss of *Tet2* derepresses myeloid lineage commitment. The deficiency of both *Dnmt3a* and *Tet2* synergistically accelerates disease development by promoting HSC self-renewal and amplification of progenitor cells. Detailed comparative studies of *Dnmt3a* and *Tet2* double-mutant HSPCs with individual gene mutant HSPCs demonstrated that Dnmt3a and Tet2 cooperatively regulate a subpopulation of genes such as *Klf4* and *Epor* [140, 141]. N*-ras*^G12D^ and *Tet2* deletion synergistically represses Spry2, a negative regulator of MAPK, thereby causing synergistic activation of MAPK signaling. Concurrence of *N-RAS* and *TET2* mutations were detected in patients with CMML or AML. Mice with compound mutations of *Tet2* and *N-ras* (*Mx1*^*Cre*^*Tet2*^fx/fx^*N-ras*^G12D^) developed accelerated CMML or AML [143, 144]. In addition, cooperation of *Tet2*^*MT*^ with *Ezh2, Asxl1,* or *Bcor* mutations in MDS/MPN, *Tet2*^*MT*^ with *Jak2*^*V617F*^* in MPN, Tet2*^*MT*^ with *Flt3*^*ITD*^, *Aml1-Eto, Pu.1*^*UREΔ/WT*^, or *Ncstn* mutations in AML, and *Tet2*^*MT*^ with *Kit*^*D816V*^ in mastocytosis have been also evaluated in mouse models (Table 2). In combination with *Tet2*^*MT*^, oncogenic kinase mutations such as *Jak2*^*V617F*^*, Flt3*^*ITD*^*,* and *Kit*^*D816V*^ promote NPM and AML development by inducing the uncontrolled proliferation of *Tet2*^*MT*^ myeloid progenitors by stimulating Akt-mTor and Jak-Stat5 signaling. In addition, *Flt3*^*ITD*^ also synergizes with *Tet*^*MT*^ in the regulation of DNA methylation and target gene expression. A distinct set of genomic loci (> 500 region including the *Gata2* gene) are hypermethylated in HSPCs with both *Flt3*^*ITD*^ and *Tet2*^*MT*^ compared to HSPCs with either mutation alone [138]. Therefore, the *Flt3*^*ITD*^ and *Tet2*^*MT*^ combination synergistically accelerates AML development. *Aml1-Eto* or *Ncstn* mutation promotes AML development in *Tet2*^*MT*^ mice by preventing myeloid progenitor cell differentiation. In addition, HMGA2, a chromatin modifier, is overexpressed in patients with MDS and AML. Mice with *Hmga2* expression and *Tet2*^*−/−*^ develop progressive phenotypes of MDS and AML. Hmga2 promotes MDS/AML development by stimulating the expression of Igf2bp2 and impairing the differentiation of *Tet2*^*−/−*^myeloid cells [146].

The cooperation of RhoA^G17V^ and Tet2^MT^ in the pathogenesis of AITL has been studied in several animal models. Over-expression of *RhoA*^*G17V*^ in CD4^+^T-cells in transgenic mice induces relatively increased TFH-cell populations accompanied by markedly reduced naive T-cell numbers. Such mice developed autoimmunity as indicated by a cellular infiltrate within the ears and tails as well as elevated serum titers of anti-double-stranded DNA antibodies and renal immune complex deposition [58]. When *RhoA*^*G17V*^ transgenic mice were crossed with *Vav-Cre*^+^*Tet2*^*fl/fl*^ mice, the compound-mutant mice developed T-cell lymphomas. Tumor cells had transcriptional signatures enriched for *mTOR*-associated genes. Transplanted tumors were responsive to the mTor inhibitor everolimus, providing a possible strategy for targeting *RhoA*^*G17V*^ lymphomas [58]. Using virally-mediated transduction of *RhoA*^*G17V*^ in *Tet2*^−/−^ HSPCs and a transplantation model, Zang et al*.*, demonstrated that *Tet2* deletion causes repression of the *FoxO1* gene, while *RhoA*^*G17V*^ promotes AKT-mediated FoxO1 phosphorylation and inactivation. RhoA^G17V^ cooperates with *Tet2* deletion to induce AITL development by collaborative inhibition of FoxO1 activity [150]. Cortes et al*.* found that Rhoa^G17V^ expression in CD4^+^ T-cells induces Tfh cell specification by increasing ICOS upregulation and stimulating PI3K and MAPK signaling. RhoA^G17V^ expression in the endogenous *RhoA* locus, together with *Tet2* loss, resulted in the development of AITL in mice [151]. Fujisawa et al*.* found that RhoA^G17V^ gains Vav1 binding ability and activates Vav1-Plcγ1-Nfat signaling by phosphorylating Vav1 in T-cell lines [161]. Collectively, RhoA^G17V^ promotes the activation of Tcr/Icos-Pi3k-mTor signaling in *Tet2*^*−/−*^ T cells, which stimulates AITL development by inducing the TFH differentiation of naive T-cells and TFH expansion.

### Potential targeted therapy for *TET2*^*MT*^ malignancies

*TET2*^*MT*^ is frequently detected in many types of hematopoietic malignancies [169]. The prognostic significance of *TET2*^*MT*^ remains a subject of debate [158]. In MDS, several studies suggested that patients with *TET2*^*MT *^tend to be associated with a lower-risk disease based on IPSS, and also show better survival and lower rates of leukemic transformation [170]. However, some other studies suggested that there is no significant difference in leukemic transformation and survival between *TET2*^*MT*^ and *TET2*^*WT*^ groups [158, 171]. In CMML*, TET2*^*MT*^ patients were older, more likely to have dysplastic blasts, a higher number of co-occurring mutations, and lower risk stratification. Importantly, *TET2*^*MT*^ was associated with a survival advantage compared to *TET2*^*WT*^ cases [172]. One study of AML patients indicated that homozygous *TET2*^*MT*^ showed significantly inferior event-free survival and a higher relapse rates compared to those with heterozygous *TET2*^*MT*^ [154].

Intensive chemotherapy is still the front-line choice of treatment for *TET2*^*MT*^ malignancies. Up until now, there have been no effective targeted therapies for *TET2*^*MT*^ malignancies. Several studies suggested that *TET2*^*MT*^ predicts the response of patients to treatment with DNA hypomethylating agents (HMA) including 5-azacytidine and decitabine [170, 173]. It is suggested that HMAs inhibit the growth of *TET2*^*MT*^ malignant cells by restoring the expression of TET2-targeted genes. However, such a conclusion is still debatable and is not confirmed by several other studies. It is most likely that the response of *TET2*^*MT*^ malignancies to HMA therapy is determined by concurrent mutations or signaling alterations. Nevertheless, 5-azacytidine might be a potential targeted therapy for *TET2*-silenced T-ALL [113]. In addition, mutant forms of several kinases such as FLT3^ITD^, cKIT^D816V^, or RhoA^G17V^ are commonly detected in many *TET2*^*MT*^ AML cases, mastocytosis, and PTCL, respectively. Studies of compound-mutant animals (including *Tet2*^*−/−*^*FLT3*^*ITD*^, *Tet2*^*−/−*^*cKIT*^*D816V*^*,* and *Tet2*^*−/−*^*RhoA*^*G17V*^) suggested that such hematopoietic malignancies are highly sensitive to a combination of HMA and a FLT3-specific inhibitor, or the multi-kinase inhibitor dasatinib [108, 173].

In addition, both restoration and inhibition of TET protein activity have been proposed for the treatment of *TET*^*MT*^ hematopoietic malignancies. Preclinical studies suggested that vitamin C (a cofactor in TET catalysis) represses *TET2*^*MT*^ cell growth by enhancing the activity of remaining WT TET2 molecules, as well as TET1 and TET3 [140, 141, 143, 167, 174]. The anti-cancer property of vitamin C can be enhanced when combined with the SIRT activator SRT1720, P300/CBP inhibitors C646 or HATi, or the HDAC I/II inhibitor trichostatin A, through regulation of the site-specific acetylation of TETs [175]. The addition of vitamin C treatment in *TET2*^*MT*^ leukemia augments the activity of residual TET-dioxygenase and enhances their sensitivity to PARP inhibition and hypomethylating agents [174, 176–178]. In a case report, an acute supraphysiological dose of vitamin C helps to eliminate chemorefractory AML cells with either *TET2*^*MT*^ or *WT1* mutation [179]. However, these conclusions have not been verified by large scale clinical studies. The optimal effective dosage of vitamin C in AML treatment has not been determined. A recent study suggested that *TET2*^*MT*^ cells might be more vulnerable to TET inhibition compared with normal HSPCs [131]. The TET inhibitor TETi76 has been tested in the preclinical setting. This study demonstrated that TETi76 restricts the growth of *TET*^MT^ leukemic cells, engineered *TET2*^−/−^ human cells, and *Tet2*^−/−^ murine HSPCs with reduced effects on normal HSPCs. TET inhibition leads to a major metabolic shift in *TET2*^*MT*^ cells, as demonstrated by significant down-modulation of c-MYC target genes. Whether TET inhibitor treatment will be beneficial to patients in the clinical setting remains to be verified.

Furthermore, in addition to regulating normal hematopoiesis and its implications in the development of malignancy, Tet2 also regulates cytokine production in macrophages and differentiation processes in both B-cells and T-cells. Thus, TETs mediate the interface between cancer and immunity [180]. Tet2 inhibition in B-cells represses the transition of GC B-cells to plasma B-cells. *Tet2* deletion in T-cells promotes the production of Th1-TNF-α and Th17-IL17 cells at the expense of Treg-IL-10 cells. Inactivation of Tet2 in the M1 type of macrophages promotes IL-6, IL-1β, and TNF-α production. In tumor models, inhibition of Tet2 in immune cells enhances antitumor immunity by reducing the functional immunosuppressive role of tumor-infiltrating myeloid cells (including MDSCs and TAM) and enhancing the tumor-killing activity of tumor-infiltrating lymphocytes (TILs) [181]. Tet2 reshapes the chromatin accessibility of several key TFs at genomic binding regions, including BATF and ETS1 in CD8^+^tumor-infiltrating lymphocytes, thereby enhancing its anti-tumor immune function and suppressing melanoma growth in vivo. Disruption of TET2 in CD19-CAR-T cells promotes anti-leukemic therapeutic efficacy [182]. However, in the B16-OVA melanoma model, TET2 in tumor cells functions as an important mediator of the IFN-γ/JAK/STAT signaling pathway. Deletion of *TET2* in tumor cells represses the expression of the checkpoint protein PD-L1 and the production of Th1-type chemokines CXCL9, CXCL10, and CXCL11. Vitamin C-stimulated TET activity can enhance TIL activity and promote the antitumor immunity induced by anti-PD1/PD-L1 treatment [183]. Thus, TET2 mediates an immune-repressive activity in T-cells and an immune-stimulatory activity in monocytes and macrophages [184]. With all of this information taken together, it is apparent that the role of TET2 in immune cells needs to be considered in future treatments for *TET2*^*MT*^ hematopoietic malignancies.

Finally, in order to develop more effective targeted medications for *TET2*^*MT*^ hematopoietic malignancies, many questions still need to be answered to better understand the pathogenesis of *TET2*^*MT*^ malignancies:

1. *Tet2* deletion in HSPCs results in dysfunction of the small-intestinal barrier and bacterial dissemination; such does not occur in myeloid cell *Tet2* deletion. Intestinal microbial-stimulated inflammatory signaling and increased interleukin-6 production are required for CMML-like disease development in *Tet2*^*−/−*^ animals. Consequently, mice with *Tet2* deletion in their myeloid progenitors (induced by LysM^Cre^) did not develop CMML-like disease. Mice with deletion of both *Tet2* and *Tet3* in HSPCs develop accelerated AML. It is important to determine whether microbe-stimulated inflammatory signaling is also required for the development of AML in *Tet2*^*−/−*^*Tet3*^*−/−*^mice. It will be also important to determine whether the deletion of both *Tet2* and *Tet3* in myeloid progenitors by LysM^Cre^ induces AML development. This will help to determine whether the AML that occurs in this model is also an HSPC-related disease and whether microbe-stimulated inflammation is required for AML development.
2. A majority of mice in a *Tet2*^*gt*^ strain (*Tet2* gene trap mice with 80% *Tet2*knockdown) developed AITL-like diseases with a long latency (∼17 months) [57]. However, most *Tet2*^*−/−*^ mice develop CMML-like disease within 1.5 years of birth, and most remaining mice develop a B-cell malignancy within 2 years. It is unknown what determines the disease identity in these animals.
3. Tet3 represses AML development in *Tet2*^*−/−*^ mice as demonstrated by *Tet2*^*−/−*^*Tet3*^*−/−*^ mice. Yet TET3 is required for the survival and proliferation of *TET2*^*MT*^ AML cells both in patients and in mouse models. The reason for such contradictory conclusions needs to be determined. Future studies also need to examine whether a specific inhibitor for certain TETs can produce better treatment effects for *TET2*^*MT*^ AML than a pan-TET inhibitor.
4. Deletion of either *Tet1/2* or *Tet2/3* in Treg cells results in downregulation of FoxP3 and impairment in the production of Treg cells. These studies suggested that all three Tet proteins collaboratively regulate Treg cell production. Future studies need to use lineage-specific deletion of all three Tets to determine how these three enzymes collaborate in HSCs, HPCs, and committed progenitor cells.
5. Loss of *TET2* in BM stromal cells (SCs) increases cell proliferation and self-renewal and enhances osteoblastic differentiation potential in BMSCs, which may, in turn, alter their behavior in supporting HSPC proliferation and differentiation. *TET2*^*MT*^ BMSCs contribute to the progression of myeloid malignancies in animal models. *Tet2*deficiency alters the BM microenvironment by facilitating the secretion of pro-inflammatory cytokines such as IL-1a, IL-5, IL-6, and CXCL5, thus favoring the expansion of leukemic progenitors [139]. It is still unknown whether TET2^MT^ or TET2 downregulation also occurs in human BMSCs and contributes to the development of malignancy in patients.
6. How can the catalytic activity-independent role of Tet2 in HSCs be attenuated by *Tet1* deletion?

## Conclusion

All three TET proteins are expressed in BM hematopoietic cells. Among them, TET2 is highly expressed in HSPCs and is involved in the regulation of lineage commitment and differentiation of HSPCs at almost all stages of the process by collaborating with key lineage-specific TFs. *TET2* deletion promotes self-renewal in HSCs and impairs differentiation of HSPCs at multiple stages, leading to a state of predisposition toward various hematopoietic malignancies. As a consequence, *TET2* mutations are commonly detected in almost all types of hematopoietic malignancies. However, a *TET2* mutation alone is not sufficient to cause hematopoietic malignancies. Rather, additional mutations are required for the transformation to hematopoietic malignancy by promoting both the survival and proliferation of *TET2*^*MT*^ HSPCs. Understanding how these concurrent mutations collaborate with *TET2*^*MT*^ in the induction of various types of hematopoietic malignancies will help us to be able to develop novel target therapies for patients with *TET2*^*MT*^ malignancies.

Although mutations of *TET1* or *TET3* are rarely detected in hematopoietic malignancies, in most *TET2*^*MT*^ malignancies, TET1 and TET3 function as tumor repressors by compensating for TET2 activity. In such tumors, TET1 and/or TET3 is (are) down-regulated due to the epigenetic methylation of enhancers/promoters. Thus, demethylating agents might prove to be useful treatments in this context. However, in many *TET2*^*MT*^ AML cases, TET3 might be required for the survival of the malignant cells. In such a situation, inhibition of TET3 activity might be a beneficial strategy for treatment. Thus, fully understanding how TET1 and/or TET3 function in regulating the survival, proliferation, and differentiation of different types of *TET2*^*MT*^ malignancies will help in the development of novel, personalized medication regimens for better overall treatment of these diseases.

## Data Availability

This is not applicable for this review.

## Abbreviations

HSPCs: Hematopoietic stem and progenitor cells
TET: Ten-eleven translocation
5mC: 5-Methylcytosine
5hmC: 5-Hydroxymethylcytosine
5fC: 5-Formylcytosine
5caC: 5-Carboxylcytosine
*TET2*^*MT*^: Mutations of *TET2* or mutant *TET2*
DSBH: Double-stranded beta-helix domain
TDG: Thymine DNA glycosylase
BER: Base-excision repair
AMPK: AMP-activated protein kinase
ARCH: Age-related clonal hematopoiesis
CHIP: Clonal hematopoiesis of indeterminate potential
TNF-α: Tumor necrosis factor-α
MDS: Myelodysplastic syndromes
MPN: Myeloproliferative neoplasm
CMML: Chronic myelomonocytic leukemia
AML: Acute myeloid leukemia
DLBCL: Diffuse large B cell lymphoma
PTCL: Peripheral T-cell lymphoma
GMPs: Granulocyte and monocyte progenitors
GC: Germinal center
AITL: Angioimmunoblastic T-cell lymphomas
PCTL-NOS: Peripheral T-cell lymphomas
CLL: Chronic lymphocytic leukemia
MM: Multiple myeloma
ALL: Acute lymphoblastic leukemia
LSC: Leukemic stem cell
IκBζ: NF-κB inhibitor zeta

## References

[1] Farlik M (2016). DNA methylation dynamics of human hematopoietic stem cell differentiation. Cell Stem Cell.

[2] Pellin D (2019). A comprehensive single cell transcriptional landscape of human hematopoietic progenitors. Nat Commun.

[3] Pastor WA, Aravind L, Rao A (2013). TETonic shift: biological roles of TET proteins in DNA demethylation and transcription. Nat Rev Mol Cell Biol.

[4] Joshi K, Liu S, Breslin SJP, Zhang J (2022). Mechanisms that regulate the activities of TET proteins. Cell Mol Life Sci.

[5] Chen Q, Chen Y, Bian C, Fujiki R, Yu X (2013). TET2 promotes histone O-GlcNAcylation during gene transcription. Nature.

[6] Zhang Q (2015). Tet2 is required to resolve inflammation by recruiting Hdac2 to specifically repress IL-6. Nature.

[7] Lazarenkov A, Sardina JL (2022). Dissecting TET2 regulatory networks in blood differentiation and cancer. Cancers (Basel)..

[8] Aivalioti MM (2022). PU.1-dependent enhancer inhibition separates Tet2-deficient hematopoiesis from malignant transformation. Blood Cancer Discov.

[9] Baessler A (2022). Tet2 coordinates with Foxo1 and Runx1 to balance T follicular helper cell and T helper 1 cell differentiation. Sci Adv..

[10] Rasmussen KD (2019). TET2 binding to enhancers facilitates transcription factor recruitment in hematopoietic cells. Genome Res.

[11] Mellen M, Ayata P, Dewell S, Kriaucionis S, Heintz N (2012). MeCP2 binds to 5hmC enriched within active genes and accessible chromatin in the nervous system. Cell.

[12] Spruijt CG (2013). Dynamic readers for 5-(hydroxy)methylcytosine and its oxidized derivatives. Cell.

[13] Hon GC (2014). 5mC oxidation by Tet2 modulates enhancer activity and timing of transcriptome reprogramming during differentiation. Mol Cell.

[14] Coulter JB (2017). TET1 deficiency attenuates the DNA damage response and promotes resistance to DNA damaging agents. Epigenetics.

[15] Kafer GR (2016). 5-Hydroxymethylcytosine marks sites of DNA damage and promotes genome stability. Cell Rep.

[16] Moran-Crusio K (2011). Tet2 loss leads to increased hematopoietic stem cell self-renewal and myeloid transformation. Cancer Cell.

[17] Tsagaratou A (2017). TET proteins regulate the lineage specification and TCR-mediated expansion of iNKT cells. Nat Immunol.

[18] Rasmussen KD (2015). Loss of TET2 in hematopoietic cells leads to DNA hypermethylation of active enhancers and induction of leukemogenesis. Genes Dev.

[19] Ko M (2011). Ten-Eleven-Translocation 2 (TET2) negatively regulates homeostasis and differentiation of hematopoietic stem cells in mice. Proc Natl Acad Sci U S A.

[20] An J (2015). Acute loss of TET function results in aggressive myeloid cancer in mice. Nat Commun.

[21] Elena C, Galli A, Bono E, Todisco G, Malcovati L (2021). Clonal hematopoiesis and myeloid malignancies: clonal dynamics and clinical implications. Curr Opin Hematol.

[22] Jaiswal S (2014). Age-related clonal hematopoiesis associated with adverse outcomes. N Engl J Med.

[23] Genovese G (2014). Clonal hematopoiesis and blood-cancer risk inferred from blood DNA sequence. N Engl J Med.

[24] Shlush LI (2018). Age-related clonal hematopoiesis. Blood.

[25] Busque L, Buscarlet M, Mollica L, Levine RL (2018). Concise review: age-related clonal hematopoiesis: stem cells tempting the devil. Stem Cells.

[26] Hormaechea-Agulla D (2021). Chronic infection drives Dnmt3a-loss-of-function clonal hematopoiesis via IFNgamma signaling. Cell Stem Cell.

[27] Hsu JI (2018). PPM1D mutations drive clonal hematopoiesis in response to cytotoxic chemotherapy. Cell Stem Cell..

[28] Abegunde SO, Buckstein R, Wells RA, Rauh MJ (2018). An inflammatory environment containing TNFalpha favors Tet2-mutant clonal hematopoiesis. Exp Hematol.

[29] Cai Z (2018). Inhibition of inflammatory signaling in Tet2 mutant preleukemic cells mitigates stress-induced abnormalities and clonal hematopoiesis. Cell Stem Cell..

[30] Meisel M (2018). Microbial signals drive pre-leukaemic myeloproliferation in a Tet2-deficient host. Nature.

[31] Abelson S (2018). Prediction of acute myeloid leukaemia risk in healthy individuals. Nature.

[32] Steensma DP (2015). Clonal hematopoiesis of indeterminate potential and its distinction from myelodysplastic syndromes. Blood.

[33] Hirsch CM (2018). Consequences of mutant TET2 on clonality and subclonal hierarchy. Leukemia.

[34] Desai P (2018). Somatic mutations precede acute myeloid leukemia years before diagnosis. Nat Med.

[35] Quivoron C (2011). TET2 inactivation results in pleiotropic hematopoietic abnormalities in mouse and is a recurrent event during human lymphomagenesis. Cancer Cell.

[36] Odejide O (2014). A targeted mutational landscape of angioimmunoblastic T-cell lymphoma. Blood.

[37] Ortmann CA (2015). Effect of mutation order on myeloproliferative neoplasms. N Engl J Med.

[38] Gurnari C (2022). TET2 mutations as a part of DNA dioxygenase deficiency in myelodysplastic syndromes. Blood Adv.

[39] Zhang T, Zhao Y, Zhao Y, Zhou J (2020). Expression and prognosis analysis of TET family in acute myeloid leukemia. Aging.

[40] Lio CW (2016). Tet2 and Tet3 cooperate with B-lineage transcription factors to regulate DNA modification and chromatin accessibility. eLife..

[41] Zhao Z (2015). Combined loss of Tet1 and Tet2 promotes B cell, but not myeloid malignancies. Mice Cell Rep.

[42] Pan F (2017). Tet2 loss leads to hypermutagenicity in haematopoietic stem/progenitor cells. Nat Commun.

[43] Li Z (2011). Deletion of Tet2 in mice leads to dysregulated hematopoietic stem cells and subsequent development of myeloid malignancies. Blood.

[44] Cimmino L (2015). TET1 is a tumor suppressor of hematopoietic malignancy. Nat Immunol.

[45] Pulikkottil AJ (2021). TET3 promotes AML growth and epigenetically regulates glucose metabolism and leukemic stem cell associated pathways. Leukemia.

[46] Schubeler D (2015). Function and information content of DNA methylation. Nature.

[47] Good CR (2017). A novel isoform of TET1 that lacks a CXXC domain is overexpressed in cancer. Nucleic Acids Res.

[48] Zhao Z (2016). The catalytic activity of TET2 is essential for its myeloid malignancy-suppressive function in hematopoietic stem/progenitor cells. Leukemia.

[49] Putiri EL (2014). Distinct and overlapping control of 5-methylcytosine and 5-hydroxymethylcytosine by the TET proteins in human cancer cells. Genome Biol.

[50] Zhang W (2016). Isoform switch of TET1 Regulates DNA demethylation and mouse development. Mol Cell.

[51] Li C (2015). Overlapping requirements for Tet2 and Tet3 in normal development and hematopoietic stem cell emergence. Cell Rep.

[52] Ma L (2022). Tet-mediated DNA demethylation regulates specification of hematopoietic stem and progenitor cells during mammalian embryogenesis. Science Adv..

[53] Dawlaty MM (2013). Combined deficiency of Tet1 and Tet2 causes epigenetic abnormalities but is compatible with postnatal development. Dev Cell.

[54] Gu TP (2011). The role of Tet3 DNA dioxygenase in epigenetic reprogramming by oocytes. Nature.

[55] Dai HQ (2016). TET-mediated DNA demethylation controls gastrulation by regulating lefty-nodal signalling. Nature.

[56] Ito K (2019). Non-catalytic roles of Tet2 are essential to regulate hematopoietic stem and progenitor cell homeostasis. Cell Rep.

[57] Muto H (2014). Reduced TET2 function leads to T-cell lymphoma with follicular helper T-cell-like features in mice. Blood Cancer J.

[58] Ng SY (2018). RhoA G17V is sufficient to induce autoimmunity and promotes T-cell lymphomagenesis in mice. Blood.

[59] Carty SA (2018). The loss of TET2 promotes CD8(+) T cell memory differentiation. J Immunol.

[60] Mouly E (2018). B-cell tumor development in Tet2-deficient mice. Blood Adv.

[61] Dominguez PM (2018). TET2 deficiency causes germinal center hyperplasia, impairs plasma cell differentiation, and promotes B-cell lymphomagenesis. Cancer Discov.

[62] Lio CJ (2019). TET enzymes augment activation-induced deaminase (AID) expression via 5-hydroxymethylcytosine modifications at the Aicda superenhancer. Science Immunol.

[63] Tanaka S (2020). Tet2 and Tet3 in B cells are required to repress CD86 and prevent autoimmunity. Nat Immunol.

[64] Orlanski S (2016). Tissue-specific DNA demethylation is required for proper B-cell differentiation and function. Proc Natl Acad Sci U S A.

[65] Shrestha R (2020). Molecular pathogenesis of progression to myeloid leukemia from TET-insufficient status. Blood Adv.

[66] Yue X, Lio CJ, Samaniego-Castruita D, Li X, Rao A (2019). Loss of TET2 and TET3 in regulatory T cells unleashes effector function. Nat Commun.

[67] Yang R (2015). Hydrogen sulfide promotes Tet1- and Tet2-mediated Foxp3 demethylation to drive regulatory T cell differentiation and maintain immune homeostasis. Immunity.

[68] Dawlaty MM (2011). Tet1 is dispensable for maintaining pluripotency and its loss is compatible with embryonic and postnatal development. Cell Stem Cell.

[69] Shide K (2012). TET2 is essential for survival and hematopoietic stem cell homeostasis. Leukemia.

[70] Kunimoto H (2012). Tet2 disruption leads to enhanced self-renewal and altered differentiation of fetal liver hematopoietic stem cells. Sci Rep.

[71] Ostrander EL (2020). Divergent effects of Dnmt3a and Tet2 mutations on hematopoietic progenitor cell fitness. Stem cell reports.

[72] Rasmussen KD (2019). TET2 binding to enhancers facilitates transcription factor recruitment in hematopoietic cells. Genome Res.

[73] Tulstrup M (2021). TET2 mutations are associated with hypermethylation at key regulatory enhancers in normal and malignant hematopoiesis. Nat Commun.

[74] Sardina JL (2018). Transcription factors drive Tet2-mediated enhancer demethylation to reprogram cell fate. Cell Stem Cell.

[75] Izzo F (2020). DNA methylation disruption reshapes the hematopoietic differentiation landscape. Nat Genet.

[76] van Oevelen C (2015). C/EBPalpha activates pre-existing and De Novo macrophage enhancers during induced pre-B cell transdifferentiation and myelopoiesis. Stem cell reports.

[77] Garcia-Gomez A (2017). TET2- and TDG-mediated changes are required for the acquisition of distinct histone modifications in divergent terminal differentiation of myeloid cells. Nucleic Acids Res.

[78] Klug M, Schmidhofer S, Gebhard C, Andreesen R, Rehli M (2013). 5-Hydroxymethylcytosine is an essential intermediate of active DNA demethylation processes in primary human monocytes. Genome Biol.

[79] de la Rica L (2013). PU.1 target genes undergo Tet2-coupled demethylation and DNMT3b-mediated methylation in monocyte-to-osteoclast differentiation. Genome Biol..

[80] Montagner S (2017). TET2 regulates mast cell differentiation and proliferation through catalytic and non-catalytic activities. Cell Rep.

[81] Mendes K (2021). The epigenetic pioneer EGR2 initiates DNA demethylation in differentiating monocytes at both stable and transient binding sites. Nat Commun.

[82] Briseno CG (2016). Distinct transcriptional programs control cross-priming in classical and monocyte-derived dendritic cells. Cell Rep.

[83] Vento-Tormo R (2016). IL-4 orchestrates STAT6-mediated DNA demethylation leading to dendritic cell differentiation. Genome Biol.

[84] Sano S (2018). Tet2-mediated clonal hematopoiesis accelerates heart failure through a mechanism involving the IL-1beta/NLRP3 inflammasome. J Am Coll Cardiol.

[85] Cull AH, Snetsinger B, Buckstein R, Wells RA, Rauh MJ (2017). Tet2 restrains inflammatory gene expression in macrophages. Exp Hematol..

[86] Florez MA (2022). Clonal hematopoiesis: Mutation-specific adaptation to environmental change. Cell Stem Cell.

[87] Madzo J (2014). Hydroxymethylation at gene regulatory regions directs stem/early progenitor cell commitment during erythropoiesis. Cell Rep.

[88] Jeong JJ (2019). Cytokine-regulated phosphorylation and activation of TET2 by JAK2 in hematopoiesis. Cancer Discov.

[89] Ge L (2014). TET2 plays an essential role in erythropoiesis by regulating lineage-specific genes via DNA oxidative demethylation in a zebrafish model. Mol Cell Biol.

[90] Qu X (2018). TET2 deficiency leads to stem cell factor-dependent clonal expansion of dysfunctional erythroid progenitors. Blood.

[91] Yan H (2017). Distinct roles for TET family proteins in regulating human erythropoiesis. Blood.

[92] Ko M (2015). TET proteins and 5-methylcytosine oxidation in hematological cancers. Immunol Rev.

[93] Cao JZ, Liu H, Wickrema A, Godley LA (2020). HIF-1 directly induces TET3 expression to enhance 5-hmC density and induce erythroid gene expression in hypoxia. Blood Adv.

[94] Suzuki T (2017). RUNX1 regulates site specificity of DNA demethylation by recruitment of DNA demethylation machineries in hematopoietic cells. Blood Adv.

[95] Huang H (2013). TET1 plays an essential oncogenic role in MLL-rearranged leukemia. Proc Natl Acad Sci U S A.

[96] Ono R (2002). LCX, leukemia-associated protein with a CXXC domain, is fused to MLL in acute myeloid leukemia with trilineage dysplasia having t(10;11)(q22;q23). Cancer Res.

[97] Tahiliani M (2009). Conversion of 5-methylcytosine to 5-hydroxymethylcytosine in mammalian DNA by MLL partner TET1. Science.

[98] Pasqualucci L (2011). Analysis of the coding genome of diffuse large B-cell lymphoma. Nat Genet.

[99] Okosun J (2014). Integrated genomic analysis identifies recurrent mutations and evolution patterns driving the initiation and progression of follicular lymphoma. Nat Genet.

[100] De Keersmaecker K (2013). Exome sequencing identifies mutation in CNOT3 and ribosomal genes RPL5 and RPL10 in T-cell acute lymphoblastic leukemia. Nat Genet.

[101] Cancer Genome Atlas Research (2013). Genomic and epigenomic landscapes of adult de novo acute myeloid leukemia. N Engl J Med..

[102] Delhommeau F (2009). Mutation in TET2 in myeloid cancers. N Engl J Med.

[103] Tefferi A (2009). Detection of mutant TET2 in myeloid malignancies other than myeloproliferative neoplasms: CMML, MDS. MDS/MPN and AML Leukemia.

[104] Langemeijer SM (2009). Acquired mutations in TET2 are common in myelodysplastic syndromes. Nat Genet.

[105] Reddy A (2017). Genetic and functional drivers of diffuse large B cell lymphoma. Cell..

[106] Sakata-Yanagimoto M (2014). Somatic RHOA mutation in angioimmunoblastic T cell lymphoma. Nat Genet.

[107] Palomero T (2014). Recurrent mutations in epigenetic regulators, RHOA and FYN kinase in peripheral T cell lymphomas. Nat Genet.

[108] Soucie E (2012). In aggressive forms of mastocytosis, TET2 loss cooperates with c-KITD816V to transform mast cells. Blood.

[109] Yao WQ (2020). Angioimmunoblastic T-cell lymphoma contains multiple clonal T-cell populations derived from a common TET2 mutant progenitor cell. J Pathol.

[110] StremenovaSpegarova J (2020). Germline TET2 loss of function causes childhood immunodeficiency and lymphoma. Blood..

[111] Quesada V (2012). Exome sequencing identifies recurrent mutations of the splicing factor SF3B1 gene in chronic lymphocytic leukemia. Nat Genet.

[112] Solary E, Bernard OA, Tefferi A, Fuks F, Vainchenker W (2014). The Ten-Eleven Translocation-2 (TET2) gene in hematopoiesis and hematopoietic diseases. Leukemia.

[113] Bensberg M (2021). TET2 as a tumor suppressor and therapeutic target in T-cell acute lymphoblastic leukemia. Proc Natl Acad Sci USA.

[114] Cheng J (2013). An extensive network of TET2-targeting MicroRNAs regulates malignant hematopoiesis. Cell Rep.

[115] Song SJ (2013). The oncogenic microRNA miR-22 targets the TET2 tumor suppressor to promote hematopoietic stem cell self-renewal and transformation. Cell Stem Cell.

[116] Ono R (2021). Tet1 is not required for myeloid leukemogenesis by MLL-ENL in novel mouse models. PLoS ONE.

[117] Wang J (2018). High expression of TET1 predicts poor survival in cytogenetically normal acute myeloid leukemia from two cohorts. EBioMedicine.

[118] Jiang X (2017). Targeted inhibition of STAT/TET1 axis as a therapeutic strategy for acute myeloid leukemia. Nat Commun.

[119] Ciccarone F (2014). Poly(ADP-ribosyl)ation is involved in the epigenetic control of TET1 gene transcription. Oncotarget.

[120] Bamezai S (2021). TET1 promotes growth of T-cell acute lymphoblastic leukemia and can be antagonized via PARP inhibition. Leukemia.

[121] Poole CJ, Lodh A, Choi JH, van Riggelen J (2019). MYC deregulates TET1 and TET2 expression to control global DNA (hydroxy)methylation and gene expression to maintain a neoplastic phenotype in T-ALL. Epigenetics Chromatin.

[122] Morin RD (2011). Frequent mutation of histone-modifying genes in non-Hodgkin lymphoma. Nature.

[123] Sun M (2013). HMGA2/TET1/HOXA9 signaling pathway regulates breast cancer growth and metastasis. Proc Natl Acad Sci U S A.

[124] Neri F (2015). TET1 is controlled by pluripotency-associated factors in ESCs and downmodulated by PRC2 in differentiated cells and tissues. Nucleic Acids Res.

[125] Song SJ (2013). MicroRNA-antagonism regulates breast cancer stemness and metastasis via TET-family-dependent chromatin remodeling. Cell.

[126] Wang Y, Zhang Y (2014). Regulation of TET protein stability by calpains. Cell Rep.

[127] Li L (2016). Epigenetic inactivation of the CpG demethylase TET1 as a DNA methylation feedback loop in human cancers. Sci Rep.

[128] Agirre X (2015). Whole-epigenome analysis in multiple myeloma reveals DNA hypermethylation of B cell-specific enhancers. Genome Res.

[129] Muller T (2012). Nuclear exclusion of TET1 is associated with loss of 5-hydroxymethylcytosine in IDH1 wild-type gliomas. Am J Pathol.

[130] Sun D (2014). Epigenomic profiling of young and aged HSCs reveals concerted changes during aging that reinforce self-renewal. Cell Stem Cell.

[131] Guan Y (2021). A therapeutic strategy for preferential targeting of TET2 mutant and TET-dioxygenase deficient cells in myeloid neoplasms. Blood cancer discovery.

[132] Chen E (2015). Distinct effects of concomitant Jak2V617F expression and Tet2 loss in mice promote disease progression in myeloproliferative neoplasms. Blood.

[133] Kameda T (2015). Loss of TET2 has dual roles in murine myeloproliferative neoplasms: disease sustainer and disease accelerator. Blood.

[134] Muto T (2013). Concurrent loss of Ezh2 and Tet2 cooperates in the pathogenesis of myelodysplastic disorders. J Exp Med.

[135] Abdel-Wahab O (2013). Deletion of Asxl1 results in myelodysplasia and severe developmental defects in vivo. J Exp Med.

[136] Iqbal J, Amador C, McKeithan TW, Chan WC (2019). Molecular and genomic landscape of peripheral t-cell lymphoma. Cancer Treat Res.

[137] Wang C (2015). IDH2R172 mutations define a unique subgroup of patients with angioimmunoblastic T-cell lymphoma. Blood.

[138] Shih AH (2015). Mutational cooperativity linked to combinatorial epigenetic gain of function in acute myeloid leukemia. Cancer Cell.

[139] Ramdas B (2020). Driver mutations in leukemia promote disease pathogenesis through a combination of cell-autonomous and niche modulation. Stem Cell Rep.

[140] Scourzic L (2016). DNMT3A(R882H) mutant and Tet2 inactivation cooperate in the deregulation of DNA methylation control to induce lymphoid malignancies in mice. Leukemia.

[141] Zhang X (2016). DNMT3A and TET2 compete and cooperate to repress lineage-specific transcription factors in hematopoietic stem cells. Nat Genet.

[142] Lobry C (2013). Notch pathway activation targets AML-initiating cell homeostasis and differentiation. J Exp Med.

[143] Kunimoto H (2018). Cooperative epigenetic remodeling by TET2 loss and NRAS mutation drives myeloid transformation and MEK inhibitor sensitivity. Cancer Cell..

[144] Jin X (2018). Oncogenic N-Ras and Tet2 haploinsufficiency collaborate to dysregulate hematopoietic stem and progenitor cells. Blood Adv.

[145] Kats LM (2014). Proto-oncogenic role of mutant IDH2 in leukemia initiation and maintenance. Cell Stem Cell.

[146] Bai J (2021). Overexpression of Hmga2 activates Igf2bp2 and remodels transcriptional program of Tet2-deficient stem cells in myeloid transformation. Oncogene.

[147] Tara S (2018). Bcor insufficiency promotes initiation and progression of myelodysplastic syndrome. Blood.

[148] Obeng EA (2016). Physiologic expression of Sf3b1(K700E) causes impaired erythropoiesis, aberrant splicing, and sensitivity to therapeutic spliceosome modulation. Cancer Cell.

[149] Lemonnier F (2016). The IDH2 R172K mutation associated with angioimmunoblastic T-cell lymphoma produces 2HG in T cells and impacts lymphoid development. Proc Natl Acad Sci.

[150] Zang S (2017). Mutations in 5-methylcytosine oxidase TET2 and RhoA cooperatively disrupt T cell homeostasis. J Clin Invest.

[151] Cortes JR (2018). RHOA G17V induces T follicular helper cell specification and promotes lymphomagenesis. Cancer Cell..

[152] Tefferi A (2009). TET2 mutations and their clinical correlates in polycythemia vera, essential thrombocythemia and myelofibrosis. Leukemia.

[153] Lin TL (2014). Clonal leukemic evolution in myelodysplastic syndromes with TET2 and IDH1/2 mutations. Haematologica.

[154] Ahn JS (2015). Adverse prognostic effect of homozygous TET2 mutation on the relapse risk of acute myeloid leukemia in patients of normal karyotype. Haematologica.

[155] Makishima H (2017). Dynamics of clonal evolution in myelodysplastic syndromes. Nat Genet.

[156] Rampal R (2014). DNA hydroxymethylation profiling reveals that WT1 mutations result in loss of TET2 function in acute myeloid leukemia. Cell Rep.

[157] Wang Y (2015). WT1 recruits TET2 to regulate its target gene expression and suppress leukemia cell proliferation. Mol Cell.

[158] Gaidzik VI (2012). TET2 mutations in acute myeloid leukemia (AML): results from a comprehensive genetic and clinical analysis of the AML study group. J Clin Oncol.

[159] Xu W (2011). Oncometabolite 2-hydroxyglutarate is a competitive inhibitor of alpha-ketoglutarate-dependent dioxygenases. Cancer Cell.

[160] Lemonnier F (2016). The IDH2 R172K mutation associated with angioimmunoblastic T-cell lymphoma produces 2HG in T cells and impacts lymphoid development. Proc Natl Acad Sci U S A.

[161] Fujisawa M (2018). Activation of RHOA-VAV1 signaling in angioimmunoblastic T-cell lymphoma. Leukemia.

[162] Vallois D (2016). Activating mutations in genes related to TCR signaling in angioimmunoblastic and other follicular helper T-cell-derived lymphomas. Blood.

[163] Küçük C (2015). Activating mutations of STAT5B and STAT3 in lymphomas derived from γδ-T or NK cells. Nature Commun..

[164] Haney SL (2016). Dnmt3a is a haploinsufficient tumor suppressor in CD8+ peripheral T Cell lymphoma. PLoS Genet.

[165] Joshi K, Zhang L, Breslin SJP, Zhang J (2019). Leukemia stem cells in the pathogenesis, progression, and treatment of acute myeloid leukemia. Adv Exp Med Biol.

[166] Redavid I (2022). Single-cell sequencing: Ariadne’s thread in the maze of acute myeloid leukemia. Diagnostics..

[167] Duchmann M, Laplane L, Itzykson R (2021). Clonal architecture and evolutionary dynamics in acute myeloid leukemias. Cancers (Basel)..

[168] Couronne L, Bastard C, Bernard OA (2012). TET2 and DNMT3A mutations in human T-cell lymphoma. N Engl J Med.

[169] Lewis NE (2020). Clonal hematopoiesis in angioimmunoblastic T-cell lymphoma with divergent evolution to myeloid neoplasms. Blood Adv.

[170] Bejar R (2014). TET2 mutations predict response to hypomethylating agents in myelodysplastic syndrome patients. Blood.

[171] Reilly B (2019). DNA methylation identifies genetically and prognostically distinct subtypes of myelodysplastic syndromes. Blood Adv.

[172] Coltro G (2020). Clinical, molecular, and prognostic correlates of number, type, and functional localization of TET2 mutations in chronic myelomonocytic leukemia (CMML)-a study of 1084 patients. Leukemia.

[173] Nguyen TB (2020). Dasatinib is an effective treatment for angioimmunoblastic T-cell lymphoma. Cancer Res.

[174] Cimmino L (2017). Restoration of TET2 function blocks aberrant self-renewal and leukemia progression. Cell..

[175] Guan Y (2020). Context dependent effects of ascorbic acid treatment in TET2 mutant myeloid neoplasia. Communications biology.

[176] Agathocleous M (2017). Ascorbate regulates haematopoietic stem cell function and leukaemogenesis. Nature.

[177] Mingay M (2018). Vitamin C-induced epigenomic remodelling in IDH1 mutant acute myeloid leukaemia. Leukemia.

[178] Zhao H (2018). The synergy of Vitamin C with decitabine activates TET2 in leukemic cells and significantly improves overall survival in elderly patients with acute myeloid leukemia. Leuk Res.

[179] Das AB (2019). Clinical remission following ascorbate treatment in a case of acute myeloid leukemia with mutations in TET2 and WT1. Blood Cancer J.

[180] Zhang Q, Casanova JL (2020). Human TET2 bridges cancer and immunity. Blood.

[181] Lee M (2021). Tet2 inactivation enhances the antitumor activity of tumor-infiltrating lymphocytes. Cancer Res.

[182] Fraietta JA (2018). Disruption of TET2 promotes the therapeutic efficacy of CD19-targeted T cells. Nature.

[183] Xu YP (2019). Tumor suppressor TET2 promotes cancer immunity and immunotherapy efficacy. J Clin Invest.

[184] Pasca S, Jurj A, Constantinescu C, Zdrenghea M, Tomuleasa C (2021). Implications of TET2 in CAR-T Cell Activity and Target Response to CAR-T Cell Therapy: Lessons Learned from T Cells. Crit Rev Immunol.

